# Genetics of Common Obesity in Children and Adolescents

**DOI:** 10.1111/nyas.70070

**Published:** 2025-10-13

**Authors:** Guadalupe León‐Reyes, Francisco J. López Alavez, Maria Elizabeth Tejero

**Affiliations:** ^1^ Laboratory of Nutrigenetics and Nutrigenomics, National Institute of Genomic Medicine (INMEGEN) Mexico City México

**Keywords:** child obesity, childhood obesity, genetic polymorphisms, single‐nucleotide polymorphisms

## Abstract

Childhood obesity is a multifactorial public health problem worldwide. Genetic variation influences the predisposition to develop obesity at early stages of life. Childhood obesity may be classified as syndromic, monogenic, or polygenic depending on the genetic component. Polygenic obesity is the most frequent, having an important interaction with environmental factors. A scoping review was conducted to identify existing literature on the association between common genetic variation and obesity‐related traits in children and adolescents. We retrieved 180 studies that were classified as genome‐wide studies, meta‐analyses targeting selected variants, and original studies with a targeted genotype approach. Most genome‐wide meta‐analyses have been conducted in Caucasian populations and have collectively identified over 100 variants in approximately 40 genes associated with obesity‐related traits. The identified genes are related to appetite regulation and energy expenditure, although in some cases, their function remains to be elucidated. Studies analyzing targeted variants have replicated some findings across populations. Some genotypes have shown varying associations, probably due to differences in study design, populations, and sample size. The effects of genetic variation on common childhood obesity require further study in diverse populations. In addition, the functional effects and clinical significance of these variants require further research.

## 1 Introduction

The prevalence of childhood overweight and obesity has increased in almost all countries. A steady increase in the number of affected children and adults has been observed in most regions of the world between 1990 and 2022 [1, 2]. According to data from the noncommunicable disease risk factor collaboration (NCD‐RisC), age‐standardized prevalence of obesity in children and adolescents between 5 and 19 years was more than 20% in girls in 21 countries (11%) and boys in 35 countries (18%). Children with obesity have a high risk of carrying excess adiposity through adulthood and of developing type 2 diabetes mellitus, hypertension, and other noncommunicable diseases at an early age, which is likely to have negative repercussions on their psychosocial and emotional well‐being [3, 4].

Childhood obesity is a complex phenotype that results from the interaction of genetic susceptibility, environmental factors, and fetal programming [5]. The most accepted definition of obesity is based on body mass index (BMI), a simple and limited proxy of adiposity. In children and adolescents, BMI is adjusted for age and sex to identify abnormal relationships between body weight and height.

There is no consensus on the definition of obesity for children under 2 years of age. For this group, analysis of length‐for‐age and weight‐for‐age charts is used as a proxy for the identification of abnormal adiposity [6]. According to the World Health Organization (WHO), overweight in children under 5 years of age is defined as weight‐for‐height greater than 2 standard deviations above the WHO Child Growth Standards median. Obesity is defined as weight‐for‐height greater than 3 standard deviations above the same median [7]. Severe pediatric (2−20 years of age) obesity is defined as BMI for age >120% of the 95th percentile or BMI at or above 35 kg/m^2^ [8].

### 1.1 Study of Genetic Factors in Childhood and Adolescent With Obesity

Initial observations suggesting the contribution of genetic factors to obesity include the aggregation of this trait in biologically related individuals, such as twins, siblings, and family members. Genetic factors influencing obesity in children and adolescents are classified into three groups: (1) syndromic forms, characterized by alterations in regions in chromosomes, causing a cluster of clinical phenotypes (a syndrome); (2) monogenic forms, caused by rare mutations in a single gene with large effects (i.e., leptin, melanocortin receptor 4, among others); and (3) common obesity, the most frequent form of obesity, which is polygenic, that is, with contribution of numerous genetic variants with small effects [9].

Syndromic and monogenic forms of obesity are relatively uncommon. Monogenic forms of obesity at present account for ∼7% of children with severe, young‐onset obesity [10]. Between 3% and 5% of children in this group have mutations in the gene *MC4R* [11].

Hereditability is a parameter that indicates that variation in a phenotype is attributable to individual genetic factors and is expressed as a percentage. Obesity and BMI can be traced to heritability more often in children than in adults, due to the shorter exposure to environmental factors [12]. Narrow‐sense heritability is the proportion of a trait variation due to additive genetic factors. The narrow‐sense heritability of BMI is 42% and common single‐nucleotide polymorphisms (SNPs) explain 23% of BMI variance [13]. The remaining 35% may be attributable to low frequency and rare variants. The contribution of genetic variants differs across BMI levels, with higher values of BMI showing a larger genetic contribution [13].

After the human genome sequence became available, new analytical methods were developed to investigate the association between variants across the human genome and obesity‐related phenotypes [13, 14]. Current studies on genetic factors influencing a given trait are performed using diverse methods for DNA analysis, depending on the study purpose. During the last two decades, studies on the genetics of obesity, and related traits in children, rapidly evolved by improving study designs and analytical methods, sample size, and variant number. Genome‐wide association (GWA) studies are widely accepted for assessing the association between thousands or millions of genetic variants, mainly SNPs, and a dichotomous or continuous trait. Thus, this approach tests many hypotheses and requires large sample sizes to achieve appropriate statistical power. Most discoveries on genetic contribution to obesity have used this approach, leading to the identification of novel variants across different genes.

The number of loci associated with childhood obesity and related traits is approximately 116 [15], while in adults, there are more than 350 genetic variants associated with similar phenotypes [14]. However, there are few studies assessing the association between common genetic variants and obesity‐related phenotypes in children and adolescents. Therefore, the present study aims to determine common genetic variation associated with polygenic obesity and related phenotypes in children and adolescents.

## 2 Methods

This scoping review followed the Preferred Reporting Items for Systematic Reviews and Meta‐Analyses (PRISMA) guidelines [16]. Database searches included PubMed and Scopus. We selected a list of search terms based on a preliminary search of the literature (see Appendix S1).

### 2.1 Inclusion Criteria

Meta‐analysis, systematic reviews, and primary studies conducted in human populations, addressing associations between genetic variants, obesity, and related phenotypes. Eligible phenotypes were BMI, body weight, waist circumference, anthropometric measures associated with adiposity, and body composition. Participants included children and adolescents 2−18 years of age. Studies had to be published in peer‐reviewed journals, in English, between 2006 and 2023. Genetic variants included SNPs using any genotyping method.

### 2.2 Exclusion Criteria

Narrative reviews, studies on syndromic, monogenic, or other rare forms of obesity or rare genetic variants (frequency <5%), as well as studies with different outcomes (i.e., insulin resistance, inflammation, appetite regulation) or not including results of an association analysis with phenotypes of interest or that the control group included adult populations.

### 2.3 Definitions

Children: Individuals aged 2−9 years old. Adolescent: According to the WHO, adolescence is the phase of life between childhood and adulthood, ranging from ages 10 to 18 [17]. Obesity: As defined by the study authors. Genetic variants: Variations in the DNA sequence. Mainly SNPs, which are identified according to a reference SNP cluster ID (rs number).

### 2.4 Classification of Studies

The studies were classified as: (1) meta‐analyses of GWAs, which are discovery studies using techniques for a genome‐wide approach (generally SNP microarrays), with no specific a priori hypothesis; (2) meta‐analyses of specific previously identified (replicated) variants, which include meta‐analyses of previously discovered genotypes or candidate variants associated with obesity that have been found and analyzed in different populations; or (3) original studies testing the association of previously discovered variants and obesity.

### 2.5 Data Extraction

Study design, sample size, age, race/ethnicity/country of origin, major variables studied, measurements of association, and study findings were extracted independently by the three authors. Studies were screened by title and abstract. Full text was analyzed as required.

### 2.6 Quality Assessment

Quality assessment of the identified GWAs and meta‐analyses of obesity in children and adolescents was conducted following the STREGA (Strengthening the Reporting of Genetic Association Studies) reporting guidelines [18].

## 3 Results

### 3.1 Literature Search Flowchart

**FIGURE 1:**
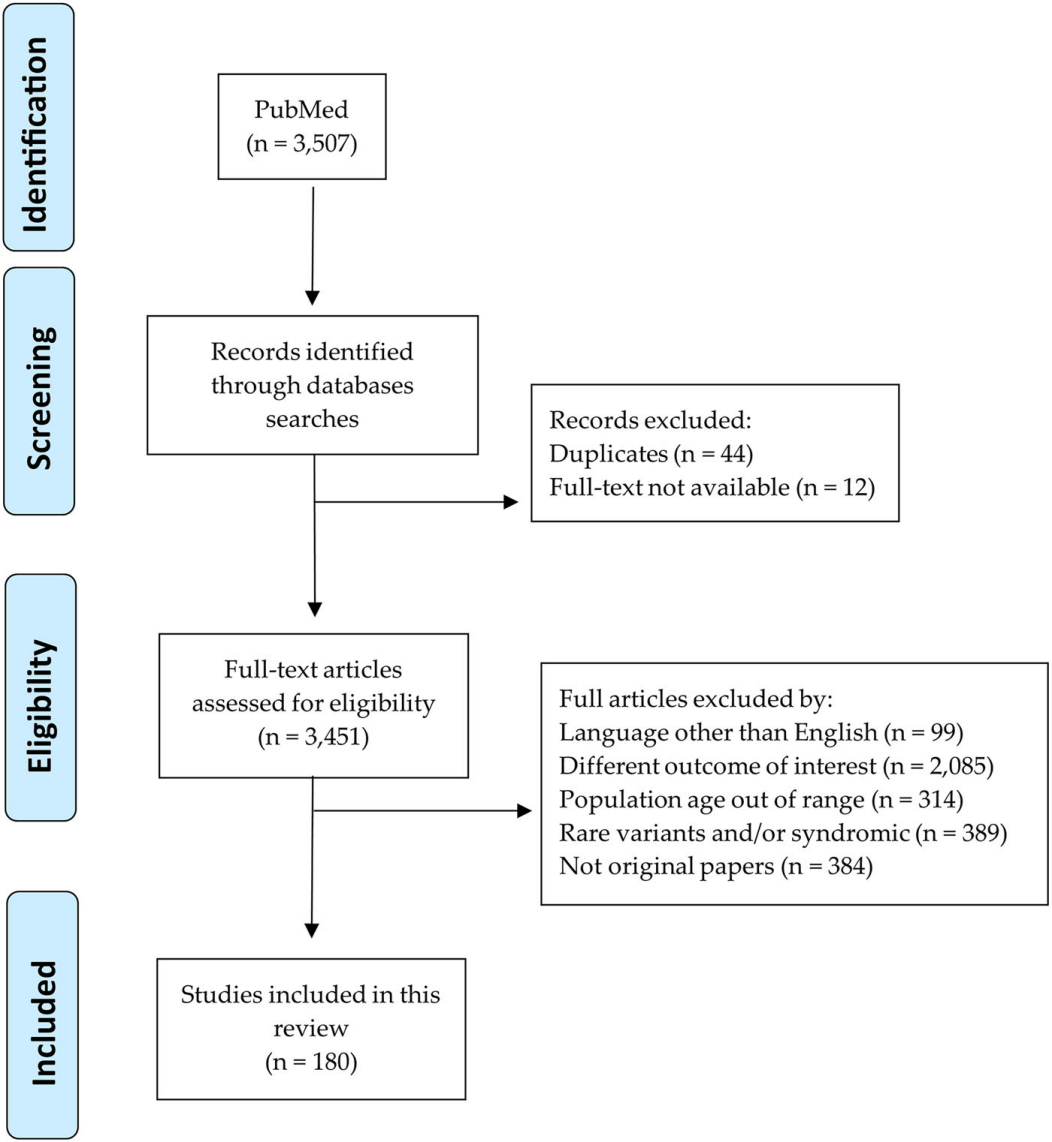
PRISMA 2020 flow chart describing the screening process.

Results of the search in scientific literature databases: The application of the search criteria identified a total of 3507 articles (Figure 1). No useful sources were identified using gray literature.

### 3.2 Findings

The present review identified 180 studies, including 16 studies using a genome‐wide approach (Table 1) [12, 19, 20, 21, 22, 23, 24, 25, 26, 27, 28, 29, 30, 31, 32, 33], 8 meta‐analyses on specific variants (Table 2) [34, 35, 36, 37, 38, 39, 40, 41], and 155 original studies analyzing the association between previously identified genetic variants and obesity‐related traits (Supplementary File: Appendix S1). The most common study designs are cohorts (*n* = 94) and case−control studies (*n* = 45), followed by cross‐sectional studies (*n* = 12) and longitudinal studies (*n* = 4). Sample size ranged from 70 to 61,111. Studies using targeted variants varied widely in sample size, as observed in Table 2 and Supplementary File: Appendix S1. Most studies were conducted in school children, or samples including children and adolescents. The most common phenotype was obesity according to the WHO definition, Centre for Disease Control (CDC) definition, local references, or nonspecified. Obesity was studied as a dichotomous variable using cut‐off points (obesity vs. nonobesity) or represented as continuous traits associated with variation in phenotypes related to body composition (i.e., BMI, body weight, body fat percentage, waist circumference, and other anthropometric measures).

**TABLE 1:** Meta‐analyses and genome‐wide studies on genetic variants associated with obesity in children and adolescents.

Study (author, year)	Study design	Sample size	Age	Country/ ethnicity	Variant (s)/genes	Genotyping	Phenotypes	Measurements of association/analysis	Study findings
Chatterjee et al. (2021) [19]	Meta‐analysis	5530 cases and 8318 controls from 14 studies	2−10 years	European ancestry	rs6887211 (*EBF*), rs7958572 (*LMRB1L*)	Genome‐wide array	Childhood obesity	Genetic analysis incorporating pleiotropy and annotation	Out of the 19 loci, one locus (*EBF1*) was novel to childhood obesity and one locus (*LMBR1L*) was novel to childhood BMI/obesity.
Vogelezang et al. (2020) [20]	Meta‐analysis	61,111 children from 41 studies 39,620 (discovery) 21,491 (replication)	2−10 years	European ancestry	25 loci, two of which were novel at *NEDD4L* and *SLC45A3*	Genome‐wide array	BMI	OR and 95% CI	The study replicated 23 loci and identified two novel loci associated with childhood obesity.
Costa‐Urrutia et al. (2020) [21]	Genome‐wide study	828 Mexican‐Mestizo children (423 boys, 405 girls)	Children and adolescents 3−16 years	Mexican Mestizos	*CERS3* and *CYP2E1*	Genome‐wide microarray	BMI % body fat	Beta values	Statistical significance was reached for *CERS3* and *CYP2E1* to BMI. Also, 11 loci in six genes (*ANKS1B*, *ARNTL2*, *KCNS3*, *LMNB1*, *SRGAP3*, *TRPC7*) reached genome‐wide significance P2E1 to BMI
Zhao et al. (2019) [22]	Meta‐analysis	18 articles using 26 studies 1229 cases 2274 controls	Children/adolescents 11.8 ± 2.9 12.7 ± 3.1	Chinese	*FTO* variants rs9939609 rs6499640 rs8050136 rs1558902	Genome‐wide array	Obesity	Pooled OR and 95% CI	The subgroup analysis showed strong association between four *FTO* SNPs (rs9939609, rs6499640, rs8050136, and rs1558902).
Bradfield et al. (2019) [12]	Trans‐ancestral meta‐analysis	13,005 cases 15,599 controls from 30 studies. Replication sample of 1888 cases and 4689 controls from seven cohorts of European and North/South American ancestry.	2−18 years	European, African, North/South American, East Asian	18 previously implicated BMI or obesity loci and a novel locus near *METTL*5	Genome‐wide array	Childhood obesity	OR and 95% CI	18 loci previously associated with BMI or obesity loci, and a novel finding.
Hwang et al. (2016) [23]	Genome‐wide study	KoCAS‐1 (*n* = 484) de novo replication in an independent population (KoCAS‐2, *n* = 1548)	10.83 ± 1.43 10.60 ± 1.36	Korean	rs10879834 in *KCNC2*	Genome‐wide microarray	Obesity‐related phenotypes	Multivariate linear regression analysis	A novel variant (rs10879834) with multiple associations for obesity‐related traits was found and replicated in an adult cohort.
Felix et al. (2016) [24]	Meta‐analysis	35,668 children in 20 studies (discovery) 11,873 children in 13 studies (replication)	3−10 years	European	Variants in genes *ADCY3*, *GNPDA2*, *TMEM18*, *SEC16B*, *FAIM2*, *FTO*, *TFAP2B*, *TNNI3K*, *MC4R*, *GPR61*, *LMX1B*, and *OLFM4*.	Genome‐wide array	Adult body mass index or childhood Obesity	Linear regression models assuming an additive genetic model adjusting for principal components if this was deemed needed in the individual studies.	In total, 15 loci reached genome‐wide significance in the joint discovery and replication analysis, of which 12 are previously identified loci.
Warrington et al. (2015) [25]	Meta‐analysis	9377 Discovery sample 3918 Replication sample	1−17 years	European ancestry	Novel loci at *FTO*, *MC4R*, and *ADCY3*	Genome‐wide array	BMI trajectories over childhood and adolescence from 1 to17 years	Association study using repeated measures.	Loci replicated and a novel.
Graff et al. (2013) [26]	Meta‐analysis	14 studies 29,880 participants	16 and 25 (late adolescents and young adults)	European ancestry	*FTO*, *TMEM18*, *MC4R*, *TNNI3K*, *SEC16B*, *GNPDA2*, and *POMC*	Genome‐wide array	BMI	Regression models adjusted for covariates.	Seven loci had statistical significant association with BMI.
Bradfield et al. (2012) [27]	Meta‐analysis	14 studies 5530 cases 8318 controls	<18 years	Children of European ancestry living in North America, Australia, European countries.	rs9568856 in *OLFM4*, rs9299 in *HOXB5*	Microarray	Obesity	OR and 95% CI	Identification of two novel loci associated with obesity.
Comuzzie et al. (2012) [28]	Cohort	815 children	4−19 years	Hispanic children in the USA	rs1056513 at *INADL*, *MATK*, *COL4A1*	GWA (Illumina Infinium)	Anthropometry, body composition	Measured genotype analysis	Revealed novel genes with unknown function in obesity pathogenesis.
Zhao et al. (2011) [29]	Cohort	728 European American children with obesity 3960 EA control 1008 African American children with obesity 2715 AA control	2–18 years	Caucasian	*MC4R* SNPs: rs9966951 rs1942880 rs12457166	Genome‐wide SNP (Illumina)	BMI ≥ 95th percentile was considered to have obesity	Adjustment for admixture in the African American cohort was carried out using logistic regression that utilized multidimensional scaling value. OR and 95% CI	Variants rs571312, rs10871777, and rs476828 (surrogates for rs17782313) yielded OR in the EA cohort of 1.142 (*p* = 0.045), 1.137 (*p* = 0.054), and 1.145 (*p* = 0.042) for obesity; however, there was no significant association with these SNPs in the AA cohort.
Wang et al. (2010) [30]	Case−control	19,822 children with obesity from KORA cohort 35,374 without obesity control 1436 from East Asian population cohort 2012 individuals from Beijing, China cohort (ALIR + CPOOA cohorts)	>17 years	Chinese	*MC4R* SNP: V103l OR = 0.87 (0.53–1.43), *p* = 0.51 ALIR study	PCR‐restriction fragment length polymorphism	Children and adolescents with an age‐ and gender‐specific BMI≥ 95th percentile were defined as having obesity, whereas those with a BMI between the 15th and 95th percentile were not defined as having obesity.	Logistic regression analysis adjusted for age, gender, and study population was used to calculate ORs of I103 carriers for obesity. OR and 95% CI	In the case–control study, no association was found between the V103I polymorphism and obesity or obesity‐related phenotypes (*p* > 0.10). In the meta‐analysis (3526 individuals from six East Asian studies), I103 carriers had a 31% lower risk for obesity (OR = 0.69, 95%, CI: 0.50–0.94, *p* = 0.02). The large meta‐analysis of the six East Asian studies and 31 studies of other ethnic groups (55,195 individuals) reported the I103 allele conferred a 21% lower risk for obesity (OR = 0.79, 95% CI: 0.71–0.88, *p* < 0.0001).
Zhao et al. (2009) [31]	Cohort	6078 children	0–18 years	European	*INSIG2* (rs17047697), *FTO* (rs8050136, rs3751812, rs7190492, rs8044769), *MC4R* (rs12970134), *TMEM18* (rs2867125, rs4854344, rs7561317), *GNPDA2* (rs13130484), *NEGR1* (rs72523773, rs72537704), *BDNF* (rs6265), *KCTD15* (rs29941), and 1q25.	Genome‐wide SNP (Illumina)	BMI	β Coefficient	Fifteen SNPs were significantly associated with BMI (*p* < 0.05), representing nine different loci with the same direction of effect as previously reported.
Frayling et al. (2007) [32]	Meta‐analysis	7477 participants	7−11 years 14 years	European whites from UK and Finland	rs9939609	Microarray	Obesity Fat mass Birth weight	Mean trait value (95% CI) by genotype	Association found from age 7 upward. Reflects a specific increase in fat mass.
Dina et al. (2007) [33]	Case control	Affected (total, three cohorts) Controls (total)	6−7 years	French: Cohort 1 French: Cohort 2 German	rs1421085 rs7817449 rs1421085 rs17817449 rs1421085 rs17817449	Microarray	Obesity	OR and 95% CI	Several potentially functional SNPs in the *FTO* region are associated with early onset and severe obesity in European populations.

Abbreviations: OR, odds ratio; SNP, single nucleotide polymorphism.

**TABLE 2:** Meta‐analyses and systematic reviews of replicated variants.

Study	Study design	Sample size	Age	Country/ ethnicity	Variant(s)/ genes	Genotyping	Phenotype	Measurements of association/analysis	Study findings
Dastgheib et al. (2021) [34]	Meta‐analysis of case/control studies	31 studies of which: (1) 13 studies with 9565 and 11,956 and controls on *MC4R* rs17782313, and (2) 18 studies with 4789 cases and 15,918 controls on *FTO* rs9939609	Children adolescents	Caucasian Asian Latin American	*FTO* rs9939606 *MC4R* rs17782313	qRT‐PCR, PCR, Sequenom, iPLEX, ARMS‐PCR, and Illumina	Obesity	OR, 95% CI	The analyzed variants are associated with obesity in Caucasian and Asian children.
Resende et al. (2021) [35]	Systematic review	5679 participants 12 studies	Children and adolescents 5−18 years	Diverse three from Asia, five from Europe, two from North America, one from Africa one evaluated multiple ethnicities	*FTO* rs9939609 *MC4R* rs17782313	Not reported	Obesity	Not calculated	Five studies founded a positive association between overweight and obesity in children and adolescents with the presence of rs17783213 and four studies with rs9939609.
Xie et al. (2020) [36]	Meta‐analysis	16 studies involving 5147 cases with overweight/obesity and 7350 controls without obesity	Children and adolescents	Latin‐America Europe East Asia (Mexico, Turkey, China, Japan, Hungary, Spain, and Germany)	*ADRB3* rs4994	PCR‐RFLP TaqMan	Overweight/ obesity	OR, 95% CI	The *ADRB3* rs4994 polymorphism could significantly increase the risk of childhood and adolescent overweight/obesity, especially for East Asia's population.
Muller et al. (2019) [37]	Association study based on a longitudinal study	Adults *n* = 3491 Children *n* = 1958	Children 11 years	Pima Indians in the USA	98 variants in genes *TMEM18* *TCF7L2 MRPS33, P4PRKD1 ZFP64, FTO*, *TAL1, CALCR, GNPDA2, CREB1, LMX1B, ADCY9, NLRC3*	Allelic discrimination	BMI z‐score	Regression	The SNP with the strongest association with BMI z‐score in childhood was rs7193144 in *FTO* (*p*=0.003). Eighty‐two variants were associated with polygenic obesity with stronger effects in childhood and adolescence. GRS was significantly associated with BMI across groups ≥5 years.
Ehtesham et al. (2019) [38]	Meta‐analysis	Case−control studies 2969 cases 2572 controls	Children (five studies) and adults	European North Africa Asian (United States, Canada, Poland, Singapore, France, Italy, Belgium)	Rare coding partial/total loss of function (LOF) in *MC3R*	TaqMan	Obesity	OR, 95% CI	Partial/total loss of function mutations in *MC3R* increases three times the risk of obesity in children.
Quan et al. (2015) [39]	Meta‐analysis	12 Case−control studies Total: 14,835 (5000 cases and 9853 controls)	Children and adolescents	Asian Amerindian Caucasian	*FTO* rs9939609	Not reported	Obesity	OR, 95% CI	*FTO* rs9939609 was significantly associated with the increased risk of obesity. Homozygous carriers are at higher risk.
Liu et al. (2013) [40]	Meta‐analysis	23 studies (11,208 cases and 35,015 controls)	Children and adolescents 6−14 years	Asian European Amerindian	*FTO* rs9939609 rs1558902 rs1421085 rs8050136	Allelic discrimination	Overweight/ obesity	OR, 95% CI	Findings suggested a positive association between *FTO* polymorphisms and overweight/obesity risk among children and adolescents.
Choquet et al. (2011) [41]	Meta‐analysis	Three independent case–control GWAS data. 3509 subjects of French and German origin.	685 French children with obesity 10.89 ± 3.27 years 685 French lean children 11.93 ± 2.27 years 487 German children with obesity 14.3 ± 3.7 years 442 German lean children	European	*CD36* rs3211867 rs3211883 rs3211908 rs1527483	SNPs extracted from GWAS	Obesity	OR, 95% CI	No association was found between variants in CD 36 with early onset obesity.

Abbreviations: *ADCY9*, adenylate cyclase 9; *ADRB3*, adrenoceptor beta 3; BMI, body mass index; *CREB1*, CAMP responsive element binding protein 1; *FTO*, FTO alpha‐ketoglutarate dependent dioxygenase; *GNPDA2*, glucosamine‐6‐phosphate deaminase 2; GRS, genetic risk score; GWAS, genome‐wide association study; *MC3R*, melanocortin 3 receptor; *MC4R*, melanocortin 4 receptor; *MRPS33P4*, mitochondrial ribosomal protein S33 pseudogene 4; *NLRC3*, NLR family CARD domain containing 3; OR, odds ratio; SNP, single nucleotide polymorphism; *TAL1*, TAL BHLH transcription factor 1, erythroid differentiation factor; *TMEM18*, transmembrane protein 18; *ZFP64*, ZFP64 zinc finger protein.

GWA studies were genotyped using different types of SNP microarrays. Regarding studies looking at targeted variants, the most common genotyping method was allele discrimination assays or arrays that allow for adjustment of the number of selected variants. Statistical analyses tested the association between an obesity‐related phenotype and SNPs using methods according to the sample size and use of covariates.

GWA studies conducted in large samples were conducted mainly in Caucasian participants. Two studies [28] used a genome‐wide approach in Mexican‐American and Mexican children, one in Korean children, with smaller sample sizes, and one in a trans‐ancestral cohort [22].

#### 3.2.1 Quality Assessment of Genetic Association Studies

Quality assessment with STREGA revealed that a total of 15 studies (94%) clearly defined the research objectives and hypotheses and specified the genetic variants analyzed using standard nomenclature. A total of 14 (88%) reported the study design and phenotypes. Also, 14 (88%) of studies reported sources of DNA, methods of genotyping, and quality control procedures. For 12 (75%), call and error rates were available. Just 11 (69%) of studies provided details on population characteristics, including ancestry, and addressed potential population stratification. Effect estimates were reported with confidence intervals in 11 (69%) of studies, and 10 (63%) disclosed the genotype frequencies. Only 7 (44%) of studies reported Hardy−Weinberg equilibrium testing in controls, and 11 (69%) clearly distinguished between primary and secondary analyses. Eleven (69%) of studies discussed potential sources of bias; 15 (94%) gave relevant evidence of findings. Overall, the studies have moderate to high quality and adhered to key STREGA recommendations, supporting the reliability of their findings (Supplementary File: Appendix S2).

### 3.3 Meta‐Analyses of Genome‐Wide Studies

GWA approaches assess thousands or millions of SNPs as markers across the whole genome in a large group of participants to obtain appropriate statistical power for association. The earliest study of childhood obesity testing a variant found in a GWA was conducted in 2007 in a German population [32]. In that year, three independent studies simultaneously reported a statistical association of genetic variants within the gene *FTO* with BMI and obesity in children and adults [32, 33, 42]. Most of the meta‐analyses identified in this review (Table 1) used data from multiple cohorts and conducted new analyses in two phases: a discovery phase that identifies statistically significant associations between genetic variants and the phenotypes of interest, followed by a validation or replication analysis in a similar sample.

As observed in Table 1, the largest meta‐analysis conducted to date [20] included 61,111 European ancestry children aged between 2 and 10 years of age. This study identified 25 loci associated with children's BMI, two of which were novel, and are located close to the genes *NEDD4L* and *SLC45A3*. The identified loci explained 3.5% of BMI variance.

Overall, these studies have replicated the associations between specific variants and obesity‐related traits such as body fat percentage, or anthropometric measures, and in some cases, novel associations were identified at new loci. To date, the most replicated variants associated with childhood and adolescent obesity are close to or harbored in genes *FTO*, *TMEM18*, *MC4R*, *FAIM2*, *NRXN3*, *FOX2P*, *ADCY3*, *SEC16B*, *TNNI3K*, *BDNF*, and *NEGR1*.

The largest multiethnic study conducted in children and adolescents included 13,500 cases and 15,599 controls [12] in the discovery phase. Participants were children and adolescents between 2 and 18 years of age, of European, North American, South American, African, and East Asian ancestry. The increase in sample size and ancestry of participants led to the confirmation of loci found in earlier studies and the discovery of novel variants. The most significant SNPs in Asian, African, and Hispanic populations were rs2540031 (*ADCY9*, *p* = 0.015), rs6567160 (*MC4R*, *p* = 8.66×10^−5^), and rs56094641 (*FTO*, *p* = 6.55×10^−6^), respectively.

### 3.4 Meta‐Analyses on Replicated Genotypes

The meta‐analyses on the association between replicated variants and obesity‐related traits (Table 2) selected eligible primary studies according to specified inclusion criteria. These studies versed on the association of one or more variants in the genes *FTO*, *MC4R*, *MC3R*, and *ADR3* and obesity‐related phenotypes in children and adolescents.

To date, the most replicated variants associated with common childhood obesity are harbored in the genes *FTO* and *MC4R* (melanocortin receptor 4). *ADRB3* encodes adrenoceptor beta 3, a protein expressed in the adipose tissue, and is involved in energy production. The rs4994 polymorphism (also known as Trp64Arg) may increase the risk of childhood and adolescent overweight/obesity, especially for the East Asia's population, as studies in different populations do not show a similar trend [36]. Although the association has been replicated across different studies, rs4994 polymorphism has not been identified by GWAs.

### 3.5 Association Studies Using Candidate Variants or Previously Discovered Genotypes

Association studies (Supplementary File: Appendix S1) tested from one to over a thousand genotypes identified in GWA studies, or proposed as candidates for association, generally having smaller sample sizes as compared to the meta‐analyses. In many populations, these are the only type of studies on the association between genetic variants and obesity‐related phenotypes in children and adolescents conducted to date.

*FTO* has shown the largest number of variants associated with obesity and BMI, accounting for a difference of approximately 0.4 kg/m^2^ [13]. Initial studies were conducted in children of European ancestry and have been extensively replicated. *FTO* is expressed in the hypothalamus, adipose tissue, and skeletal muscle [32] and encodes for a nuclear protein of a Fe(II)‐ and α‐ketoglutarate‐dependent dioxygenase involved in the demethylation of *N* ^6^‐methyladenosine (m⁶A) and *N* ^6^,2′‐O‐dimethyladenosine (m⁶Am) on RNA [43]. These demethylations regulate critical processes such as RNA stability, splicing, and translation, influencing energy metabolism, adipogenesis, and appetite control [32]. In addition, functional studies suggest that FTO may regulate downstream genes such as *IRX3*, which is involved in adipogenesis and energy balance, promoting the hypothalamic leptin resistance induced by a high‐fat diet [44]. Moreover, FTO regulates alternative splicing of adipogenic regulatory factor RUNX1T1 (runt‐related transcription factor 1), modulating preadipocyte differentiation [45].

A meta‐analysis on the variation in *FTO* (SNPs rs9939609, rs1421085, rs1558902, rs8050136) and obesity‐related phenotypes in children and adolescents from 23 studies [40] included European, Asian [46], and Amerindian [47] participants showing replication across studies conducted in diverse countries and ancestries, study designs, sample sizes, and analytical models [48]. These analyses showed a similar effect on the risk for obesity across different populations, although some inconsistent findings have been reported [49]. The included meta‐analyses show a statistically significant association of variants in this gene with overweight, obesity, and/or higher BMI across different populations and age groups [34].

The second gene containing variants with replicated associations with obesity and related phenotypes is *MC4R*, which encodes for a receptor specific to the heptapeptide core common to adrenocorticotropic hormone and α‐, β‐, and γ‐MSH. This is a highly polymorphic gene, with many rare and common variants, including some associated with common obesity and/or with large effects on BMI, causing monogenic obesity. Given their low frequency, they do not have a significant contribution to common obesity [50]. Common variants (> 5%) in this gene have modest effects on BMI. MC4R belongs to the G‐protein‐coupled receptor (GPCR) family and is expressed in many tissues, including the hypothalamus [45]. Activation of MC4R by its endogenous ligand, α‐melanocyte‐stimulating hormone (α‐MSH) derived from pro‐opiomelanocortin (POMC), leads to the stimulation of adenylate cyclase, increasing cyclic adenosine monophosphate (cAMP) levels, which in turn suppresses food intake and enhances energy expenditure. MC4R encodes a GPCR critical in the central regulation of energy balance, appetite, and satiety through the hypothalamic leptin‐melanocortin pathway [48, 51, 52]. Pathogenic mutations in *MC4R* are found in up to 5% of cases of severe childhood obesity, and up to 0.3% of the general population [53]. Most variants in this receptor decrease or cause loss of function, while a few of them may increase their function. These effects correlate with appetite regulation.

The gene transmembrane protein 18 (*TMEM18*) is expressed in several regions of the brain, including the hypothalamus, which is responsible for the regulation of feeding behavior, and has a key role in energy regulation and obesity pathogenesis. *TMEM18* expression is downregulated in the adipose tissue of children with obesity, correlating with impaired adipocyte formation and insulin resistance. At the molecular level, TMEM18 is at the nuclear membrane and exhibits sequence‐specific DNA‐binding activity, suggesting a potential role in transcriptional regulation [54, 55]. This protein is involved in transcriptional repression, and the association with obesity is unclear. SNP variants in this gene have been associated with childhood and adult obesity across different studies and populations [56, 57]. According to a recent study, *TMEM18* is an upstream regulator of *PPARG* signaling driving healthy adipogenesis, which is dysregulated with adipose tissue dysfunction and obesity [58].

The gene SEC 16 homolog B endoplasmic reticulum export factor (*SEC16B*) is also expressed in the brain and is involved in the organization of transitional endoplasmic reticulum sites and protein export. Functional studies have elucidated its role in lipid metabolism and energy intake in animal models [59, 60]. A recent study [61] showed that *SEC16B* plays an important role in chylomicron metabolism, which may shed light on the association between variants in this gene and obesity in humans.

Other genes containing SNP variants associated with obesity and related traits in children and adolescents are the Fas apoptotic inhibitory molecule 2 (*FAIM2*) and neurexin 3 (*NRXN3*). The first one has ubiquitous expression, although its biological activity is unknown. *FAIM2* encodes a membrane‐associated protein that inhibits Fas‐mediated apoptosis, particularly in neuronal cells. GWA studies have identified SNPs near FAIM2 being associated with increased BMI and obesity risk. Although its biological function is not yet fully known, its expression is regulated by nutritional status, with increased expression observed in the hypothalamic arcuate nucleus during food deprivation [62].

*NRXN3* encodes neurexin‐3, a synaptic cell adhesion molecule involved in neuronal communication and synaptic plasticity. GWA studies have identified variants in *NRXN3* associated with increased waist circumference, BMI, and obesity. Functional studies have shown that *NRXN3* expression in the hypothalamic paraventricular nucleus is upregulated in response to metabolic stressors such as fasting and cold exposure [63]. These findings suggest that NRXN3 influences obesity through central nervous system pathways, potentially affecting reward‐related behaviors and metabolic processes. This protein functions in the nervous system as a receptor and a cell adhesion molecule [29].

The gene forkhead box 2 (*FOXP2*) is ubiquitously expressed and encodes for a transcription factor required for the proper development of speech and language. Its association with obesity is unclear [37, 48]. Emerging evidence suggests that *FOXP2* may influence metabolic processes related to obesity by increasing BMI, particularly in individuals with neuropsychiatric conditions such as schizophrenia. Furthermore, epigenetic analyses have revealed differential DNA methylation patterns of *FOXP2* in adipose tissue of individuals with obesity [64]. These findings suggest that *FOXP2* may contribute to obesity through mechanisms involving both neural regulation of energy balance and peripheral metabolic pathways. In this context, changes in methylation at single CpGs within *FOXP2* (cg26580413) were correlated with changes in weight (*FOXP2*, *R* = 0.85), and copy number variants in *FOXP2* have been associated with childhood obesity and nominally associated with obesity in a familial case−control study [64, 65].

The gene adenylate cyclase 3 (*ADCY3*) produces a membrane‐associated enzyme that catalyzes the formation of the secondary messenger cAMP. This gene colocalizes with *MC4R* at the primary cilia of a subset of hypothalamic neurons implicated in body‐weight regulation. SNPs in *ADCY3* were associated with childhood obesity and associated traits at a genome‐wide significance level [66]. ADCY3 plays a central role in the leptin–melanocortin pathway, a critical system for controlling hunger and energy expenditure. Dysfunction of this pathway, such as through ADCY3 loss‐of‐function mutations, has been associated with severe early onset obesity, hyperphagia, insulin resistance, and dyslipidemia [24]. Moreover, mutations in this gene can lead to leptin resistance, impairing the brain's ability to suppress appetite after food intake. Therefore, ADCY3 is an essential component of central mechanisms governing energy balance and represents a potential therapeutic target for obesity and related metabolic disorders [24, 29].

TNNI3 interacting kinase (*TNNI3K*) is expressed in the heart, and its function is unknown. Some SNP variants have been related to BMI in childhood and adolescence [9, 24]. These associations have been observed across diverse populations, including European, Korean, and Mexican cohorts. Furthermore, interactions between *TNNI3K* variants and other genetic loci, such as *ITIH4*, have been linked to childhood obesity risk, suggesting that *TNNI3K* may influence obesity through complex genetic networks. Although the precise mechanisms remain unclear, these findings highlight *TNNI3K* as a gene of interest in obesity research [29, 67].

Variants in the gene brain‐derived nuclear factor (*BDNF*) have been consistently associated with childhood BMI and obesity. This gene is widely expressed in the brain, predominantly in the hypothalamus, and plays relevant roles in neuronal plasticity and in regulating appetite and body weight. Recently, *BDNF* has been associated with the melanocortin signaling pathway, suggesting a role in appetite regulation [9, 24]. In humans, rare mutations or deletions in *BDNF* are associated with early onset obesity, hyperphagia, and cognitive impairments. Circulating BDNF levels have been studied as potential biomarkers for obesity, with some studies reporting decreased levels in individuals with obesity, although findings are inconsistent. Additionally, physical exercise has been shown to elevate BDNF levels, suggesting a therapeutic opportunity for obesity management [68, 69].

The gene neuronal growth regulator‐1 (*NEGR1*), expressed only in the brain, is a member of the immunoglobulin superfamily, with a role in cell adhesion and neurite outgrowth in the developing brain. The role of this gene in the development of obesity remains to be discovered [9, 24, 29].

## 4 Discussion

GWA studies have identified a variety of loci related to BMI and adiposity traits in pathways regulating appetite, energy balance, and adipose tissue function [14]. Polygenic obesity has an inheritance pattern similar to other complex traits and diseases, such as type 2 diabetes mellitus or cardiovascular disease. Individuals with common obesity may carry multiple genetic susceptibility variants, with a minor effect on weight [3]. Studies performed with children and adolescents have identified novel variants that had been undetected in adult populations. This can be explained by the higher heritability found in younger age groups and shorter exposure to environmental factors as compared to adults [22, 24, 25, 27, 31, 70, 71].

Heterogeneity in sample size, study design, phenotypes, and genotyping methods was a key limitation of the included studies. For instance, the identified GWAs analyzed different phenotypes, including obesity as a dichotomous trait, using different definitions, which may impact the prevalence of obesity and overweight [72], different phenotypes, such as BMI z‐score, body fat %, among others, which may lead to inconsistent findings, although related traits may share genetic variants.

Differences in genotyping across studies arise from using diverse microarrays, which vary in SNP content and number, coverage, imputation quality, and other characteristics that may limit the comparability of findings across studies [73]. Many studies lacked standardized criteria in the control group or did not consider population stratification, generating a potential bias.

Zhou et al. [15] conducted a scope review on GWA studies on childhood obesity and related phenotypes, finding SNPs shared by obesity, BMI, and adiposity. Most of the discovery studies addressing the genetic contribution to common obesity have analyzed European or Caucasian populations. Very few GWA studies were conducted in non‐Caucasian children. One of the identified studies was conducted in Mexican American children, one in Mexican children, and one in Korean. Findings in these studies replicated some previously identified loci, some biologically plausible genes, and novel variants [21, 23, 28]. The overrepresentation of European or Caucasian populations restricts the generalizability of the findings of the scoping review. Genetic findings, as allele frequencies or the genetic architecture, vary significantly across ethnic groups. Investigating genetic risk in admixed populations, for example, African or Amerindian ethnic groups, is essential for discovering ancestry‐specific risk variants. In this sense, Zhu and Wang [74] described ancestry mapping as an efficient methodology for assessing differences in local ancestry. Ancestry mapping allows detecting genetic associations with complex traits with less bias from multiple testing compared to traditional GWAS. Additionally, Martin et al. [75] examined global and local ancestry adjustments in genetic association studies. Authors reported improved power with ancestry correction for estimating complex disease effects. These approaches are efficient tools for identifying relevant loci in admixed‐race individuals, which could provide a more accurate and representative understanding of the genetic basis of obesity. According to Loos and Yeo [14], loci identified in one ancestry demonstrate directionally consistent associations across other ancestries, even though effect sizes and allele frequencies may differ. Body fat distribution is also associated with ethnicity [76]. Asian populations have lower obesity rates; however, they have a higher risk for type 2 diabetes [77]. Studies conducted in non‐European ancestry populations have identified novel loci with a small contribution to common obesity.

### 4.1 Ethnicity and Other Factors Associated With Obesity

The obesity prevalence varies in different countries and ethnic groups. The obesity rate in European and Asian populations is less than 35%, while it is as high as 50% in Pima Indian and Pacific Island populations [78]. In the United States, obesity is 10–40% higher among Native Americans, Native Hawaiians, Hispanics, and Blacks, as opposed to their White counterparts [79]. Many studies report information on ethnicity as a proxy of ancestry. As aforementioned, the prevalence of childhood and adult obesity is increasing in most countries, regardless of the genetic background. Common obesity is influenced by factors that also vary across populations, such as education, culture, and socioeconomic conditions [80]. Hruby and Hu discussed the role of socioeconomic and environmental factors such as income, education, and built environment as risk factors for obesity. In some cases, these factors are strongly associated with ethnicity, which may contribute to the differences in obesity prevalence across different countries or ethnicities within a country [80]. In the United States, non‐Hispanic black women and children, Mexican‐American women and children, low‐socioeconomic status (SES) African‐American men and white women and children, Native American, and Pacific Islanders of low socioeconomic status are disproportionately affected by obesity at all ages. Meanwhile, some minority groups, such as Asian American, have a lower prevalence of obesity [81]. Gene−environment interactions are critical in modulating genetic risk, highlighting the importance of lifestyle, socioeconomic factors, and early life exposures [82].

### 4.2 Interaction Between Variants and Additive Effects

The combined effects of common variants or epistasis in *FTO* and *MC4R* have been investigated, showing significant effects on obesity and related traits in children and adolescents [83]. A recent systematic review on this interaction included 12 studies showing inconsistent results [35]. While individual variants confer modest effects, their cumulative influence, estimated through genetic risk scores or GRSs, is powerful in evaluating an individual's genetic risk for obesity. Different GRS (also called polygenic risk scores) have been investigated for obesity in children, generally using variants with small effects and replicated associations [84]. The largest association study conducted to date on the genetics of childhood obesity (*n* = 61,111) developed a GRS based on 25 SNPs showing significant association with BMI [85]. Craig et al. [86] highlighted the potential of GRSs to predict differential responses to lifestyle interventions; they analyzed data from 27 studies, including 7928 children and adolescents with overweight or obesity. Some SNPs were associated with BMI and body composition following dietary and physical activity interventions. Likewise, Yengo et al. [87] integrated GRSs with environmental and behavioral data, increasing their predictive value. Therefore, the utility of GRSs extends beyond prediction; they are promising instruments for explaining gene−environment interactions and may guide precision public health strategies for early diagnosis, personalized intervention, and long‐term obesity prevention in children and adolescents [86]. Most of the GRSs have been developed and tested in participants of European ancestry, and their use in different populations requires validation [88]. These efforts could ultimately lead to developing predictive models and effective prevention strategies to mitigate the global burden of pediatric obesity. In a broader context, this knowledge may support the development of evidence‐based public health policies to reduce the incidence and long‐term consequences of pediatric obesity [84].

### 4.3 Effects of Genetic Variants on the Response to Obesity Treatments in Children

The effect of genetic variants related to obesity in children and adolescents has been tested in very diverse intervention studies aimed to induce weight loss in children. A systematic review and meta‐analysis of these studies identified a few variants with statistically significant effects on the response to lifestyle interventions (rs12429545, rs10926984, and rs543874). The effect sizes and 95% confidence intervals (CI) are −0.002 (−0.004, −0.001), −0.152 (−0.198, −0.107), and −0.002 (−0.003, −0.001) [89]. This study concluded that gene variants appear to play a minor role in lifestyle‐based obesity interventions in children and adolescents. More high‐quality randomized controlled trials are needed to examine these effects [89].

According to studies on the BMI trajectories from childhood into adulthood, the contribution of genetic variants to the risk of obesity remains relatively stable over the life‐course, although some studies suggest that the effect of some SNPs on obesity‐related traits may vary throughout a person's life [25, 90, 91]. Such is the case of variants within *FTO* and *MC4R* [92].

Understanding the genetic basis of obesity in children requires a comprehensive approach that considers SNP‐based associations and non‐SNP genetic mechanisms such as epigenetic modifications, copy number variations (CVNs), and gene expression regulation. Epigenetic mechanisms regulate gene expression at the transcription and translation levels. They are sources of phenotypic variation and are major mediators of the response to the environment. CVNs are DNA sequences of approximately 1 kb, containing deletions or duplications. The regions showing association with obesity‐related phenotypes are located at 11q11 (*OR4P4*, *OR4S2*, *OR4C6*), 1p21.1 (*AMΥ1*), 10q11.22 (*NPY4R*), 10q26.3 (*CYP2E1*), 16q12.2 (*FTO*), 16p12.3 (*GPRC5b*), and 4q25 [82] and have been associated with adult obesity as well [93, 94]. The mechanisms underlying these associations are beyond the scope of this review. During the last two decades, studies on the genetics of obesity, and related traits in children, rapidly evolved using more appropriate designs and analytical methods, significantly increased in sample size and variant number. Recent studies have investigated more informative phenotypes, such as changes over time, and assessed complex effects such as the integrated contribution of genetic variants to the risk for disease and response to treatments.

### 4.4 Strengths and Limitations

The main strength of the present study is the review of diverse studies addressing the association between genetic variations and obesity‐related traits in children and adolescents from different ancestries and regions around the world. The main limitations identified in this group of studies are the heterogeneity of analytical strategies, limited phenotypes, and the underrepresentation of non‐Caucasian populations in association studies and those aimed to investigate the clinical relevance of variants as predictors for disease or response to interventions. We also limited eligibility criteria to 2 years of age and older; the study of younger children was beyond the scope of this work.

## 5 Summary

There are approximately 135 loci in 12−15 genes showing consistent and reproducible association with common obesity in children and adolescents. Most of the identified genes are expressed in the brain, although their specific biological role and the contribution of their variants to obesity remain to be elucidated. Most of the identified variants have been associated with adult obesity, suggesting that the genetic contribution to body composition is overall stable throughout life. A very small number of variants have been associated only with childhood obesity. As observed in this review, investigation in this field has evolved rapidly using advanced technology and research strategies; however, most studies addressing the genetic contribution to obesity were conducted in Caucasian populations. Few large, properly powered studies were performed in other populations using high‐throughput technology (genome‐wide analyses). Studies in diverse populations such as African‐derived groups, Latin American, and South Asian using genome‐wide approaches are needed. Another important requirement for future research is the harmonization of genotyping and phenotyping methods. In addition, analytical methods that integrate the effects of diverse variants (i.e., interaction, additive effects, counteracting effects, etc.) and their interaction with environmental factors are under extensive research. Therefore, future research should prioritize harmonizing methods, diagnostic parameters, and measurement protocols, thus enhancing data comparability, bolstering the reliability of meta‐analyses, and facilitating the identification of consistent and clinically relevant genetic markers.

## Author Contributions

G.L.‐R. and F.J.L.A. conducted the literature search review and extracted data. M.E.T. designed the study, conducted the search, extracted data, and wrote the manuscript.

## Conflicts of Interest

The authors declare no competing interests.

## Supporting information



Supplementary Material: Appendix 1

Supplementary Material: Appendix 2

## Data Availability

Research data are not shared.
