# Supplementary material for: Genetics of Common Obesity in Children and Adolescents
Source: Ann N Y Acad Sci. 2025 Oct 13;1553(1):34–49. doi: 10.1111/nyas.70070 (PMC12645270; doi:10.1111/nyas.70070)
Supplement: Supplementary file 1 — Supplementary Material: Appendix 1 [file NYAS-1553-34-s001.docx]

**Appendix 1.** Association studies using candidate variants or previously discovered genotypes.

| **Study** | **Study Design** | **Sample size** | **Age** | **Country/ ethnicity** | **Variant(s)/ genes** | **Genotyping** | **Phenotype** | **Measurements**  **of association/**  **analysis** | **Study Findings** |
| --- | --- | --- | --- | --- | --- | --- | --- | --- | --- |
| Adiyeva M. et al., 2023.^1^ | Case-control | 184 adolescents | 15 to 18 y | Kazakhstan | *AGTR1*  rs5186  *AGТ*  rs4762  *LPL*  rs328  *ADRB2*  rs1042714 | Allelic discrimination | Ratio of waist volume/hip volume (WV/HV) as indicators of adiposity distribution | OR, 95% CI | The rs328-G polymorphism on *LPL* gene reduces the risk of developing abdominal obesity compared to the C allele. The C/G genotype reduces the risk of developing abdominal obesity. |
| Manco L. et al., 2022.^2^ | Cohort | 637 children | 3–11 y | Portugal | *SLC6A4*  5-HTTLPR STin2  *MAOA*  VNTR | PCR-based methods | BMI Z-score, WHtR, sum of skinfolds and WC | OR, 95% CI  β, 95% CI | Carriers of the two *SLC6A4* SNPs revealed in girls significant protective effects against BMI (β=-0.615), BMI Z-score (β=-0.251), WC (β=-1.4) and WHtR (β=-0.008). |
| Reuter E. et al., 2021.^3^ | Cohort | 355 children | 7 to 15 y | Brazil | *FTO*  rs9939609 | Allelic discrimination | Waist circumference, waist-to-height ratio, BMI, and body fat % | RR, 95%CI | A statistically significant association between the  rs9939609-A *FTO* (homozygous or heterozygous) was associated with a higher risk of increased abdominal fat, as determined by WC and WHtR. |
| Walia G. et al., 2021.^4^ | Cohort | 208 children | 14.4±1.7 y | India | *MC4R*  rs17782313 | PCR-RFLP | BMI, WC, hip circumference, weight | β ± SE | The *MC4R* -rs17782313 predicted increased body weight (β:0.15, S.E ± 0.076, P=0.043) among children. |
| Molina-Luque R. et al., 2021.^5^ | Cohort | 220 children | 6 to 11 y | Chile | *FTO*  rs9939609 | RT-PCR | BMI and abdominal obesity | Prevalence ratios, 95%CI, β-coefficient. | The *FTO* rs9939609 -AA was associated with an increase in waist circumference (PR:1.53, P=0.001) in Chilean children. |
| Kroll C. et al., 2021. ^6^ | Cohort | Follow-up 1:  211 children  Follow-up 2:  187 children | Follow-up 1: 4-5 y  Follow-up 2: 6 y | Brazil | *ADIPOQ*  rs2241766  *LEP*  rs7799039 | PCR-RFLP | BMI | RR, 95% CI | The *ADIPOQ*-rs2241766 TG/GG was associated with a higher risk of excess body weight in the first 6 years (RR: 1.25). BMI increased over the years according to the presence of the TG/GG genotype in females. The *LEP*-rs7799039 was not associated with body weight. |
| Enciso-Ramírez M. et al., 2021.^7^ | Cohort | 63 children | 7 to 12 y | Mexican | *CD36*  *rs1761667* | PCR-RFLP | Weight, %body fat, waist circumference,  Hip, waist-hip ratio and BMI | OR, 95% CI | The G allele on CD36 was associated with high BMI z-score in children (OR=2.43, p=0.02). |
| Raskiliene A. et al., 2021.^8^ | Cohort | 1,082 adolescents | 12-13 y | Lithuania | *MC4R*  rs17782313  *LEP*  rs7799039  *LEPR*  rs1137101 | RT-PCR | BMI, skinfold | OR, 95% CI | BMI was not associated with any variant. *MC4R* rs17782313-C carriers had higher values of subscapular skinfold thickness than TT-carriers. *LEP* rs7799039-AG was associated with lower values of triceps skinfold thickness. |
| Quevedo F. et al., 2021.^9^ | Cohort | 382 children and adolescents | 6 to 17 y | Brazil | *FTO*  rs9939609 | RT-PCR | waist circumference, BMI and body fat percentage. | OR, 95% CI | The rs9939609-AA genotype was associated with higher waist circumference in schoolchildren (β=4.40; p=0.048). However, for BF%, the genotype association was found in the upper PI tertile (β=7.35; p=0.040). |
| Kulaeva E. et al., 2021.^10^ | Case - control | 367 children and adolescents (65 no obesity and 302 with overweight) | 4 to 18 y | Russia | *FTO*  rs99305069  *PON1*  Gln192Arg  *LIPC*  -250G>A  *LPL*  Ser447Ter | SNP-Express reagent kit | BMI | OR, 95% CI | A two-locus genotype FTO AT/LPL SerTer associated with a reduced risk of overweight in children was identified (X^2^=4.88, p = 0.027; OR = 0.35; 95% CI 0.15–0.83). |
| Maguolo A. et al., 2020.^11^ | Cohort | 1,649 children and adolescents | 10.9±2.6 y | Italy | *FADS*  rs1535  *ELOVL2*  rs2236212 | Allelic discrimination | BMI | β ± SE | A positive association between zBMI and the rs2236212-C in *ELOVL2* was found (β=0.053, p=0.028. Carriers of rs1535-C in *FADS2* had lower zBMI and BMI compared to CA/AA carriers. A negative association between rs1535 and BMI was found (β=−0.028). |
| García-Rodríguez M., 2020.^12^ | Cohort | 477 children | 9 to 12 y | Mexico | *HNFA*  *rs1800961* | Allelic discrimination | Waist circumference | OR, 95% CI | Carriers of the *rs1800961-*risk allele show higher abdominal obesity (OR=1.20; 95% CI 1.09–4.50; P=0.029). |
| López-Rodríguez G. et al., 2020.^13^ | Cohort | 750 children | 4 to 13 y | Mexico | *MC4R*  *Rs17700144*  *Rs17782313*  *LEP*  *Rs7799039*  *LEPR*  *Rs1137101*  *FTO*  *Rs8050136*  *Rs9939609*  *Rs3751812* | Allelic discrimination | BMI | OR, 95% CI | The *FTO* (rs8050136, rs9939609) and *MC4R* (rs17782313) genotypes were significantly associated with obesity (BMI > 2Z) in boys (OR=1.89, P=0.04, OR=3.3, P=0.006 OR=3.11, p=0.04, respectively). |
| Costa-Urrutia P. et al., 2020.^14^ | Cohort | 461 children  (187 with overweight or obesity and 274 without obesity ). | 4 to 13 y | Brazil | *ACTN3*  R577X  ACE I/D | PCR | BMI | OR, 95% CI | Carriers of both polymorphisms shown a clear association with severe obesity in schoolchildren. |
| Meng Y. et al., 2020.^15^ | Cohort | 121 children | 9 to 10 y | USA | *CLOCK*  *rs1801260*  *FTO*  *rs9939609* | Allelic discrimination | BMI z-score | β, 95% CI | The associations between BMI z-scores and the two polymorphisms in CLOCK and FTO did not reach statistically significance. |
| Ulloa N. et al., 2020.^16^ | Cohort | 361 children | 6 to 11 y | Chile | *FTO*  rs9939609 | PCR | BMI z-score, body weight, WC | β, 95% CI | The rs9939609 was associated with the increase of 2.47 kg in body weight, 1.06 kg/m^2^ in BMI, 2.55 cm in WC and 1.98% in body fat, also the WC/height index was associated FTO. |
| Carrillo-Venzor M. et al., 2020.^17^ | Cohort | 412 teenagers | 12 to 18 y | Mexican  (Mestizo  Tarahumara  Mennonite) | *PPAR-γ2*  Pro12Ala  *PPAR-δ*  +294T/C | PCR | BMI, BMI-z score, WHR | OR, 95% CI | Teenagers with PPAR-γ2 G allele showed a greater risk for WHR (OR:1.79), whereas they showed lower BMI Z-score with Pro12Ala (OR:0.46) and increase with +294T/C (OR:1.78). |
| Justice A. et al., 2019.^18^ | Cohort | 577 at 5y,  770 at 10y  545 at 16y | 5, 10 and 16 y | Chile | 18 SNPs in SEC16B  ADCY3  FAIM2  OLFM4  FTO, GPR61  TNNI3K  TMEM18  GNPDA2  TFAP2B  ELP3, MC4R  LMX1B  RAB27B | Illumina Infinium Multi-Ethnic Global-8 array | BMIz score | β, SE | At 5 years, only *SEC16B* was associated with BMI-z (β:0.126). The SNPs associated at 10 y were at the *SEC16B* (β:0.185), *ADCY5* (β:0.099), *FAIM2* (β:0.116), *OLFM4* (β:0.100) and *FTO* (β:0.119); while at 16 y the *SEC16B* (β:0.164) and *FTO* (β:0.177) had a statistically significant association. |
| Manco L. et al., 2019.^19^ | Cohort | 440 children and adolescents  319 normal weight  98 with overweight  23 with obesity | 3 to 11 y | Portuguese | *FTO*  *rs9939609* | Allelic discrimination | BMI, BMI z-score, waist circumference and waist-to-heigh ratio | OR, 95% CI and β, SE | The rs9939609-A was associated with BMI (P=0.029), BMI Z-score (P=0.017), WC (P=0.016), and WHtR (P=0.019), and marginally associated with overweight/obesity (OR: 1.37; P=0.049). When stratified by sex, it showed marginal or significant associations with BMI (P=0.08), BMI Z score (P=0.07), WC (P=0.005), WHtR (P=0.02), and overweight/obesity (OR: 1.52; P=0.064) in girls but not in boys (P >0.05). |
| Pascual-Gamarra J. et al., 2019.^20^ | Cross-sectional study | 1,057 adolescents | 12 to 18 y | Europe | *18 SNPS in*  *UCP1*  *UCP2*  *UCP3* | GoldenGate technology | Weight, waist, BMI, skinfold thickness | OR, 95% CI | The *UCP1* rs653699-C allele was associated with a lower risk of overweight (OR: 0.72; 95% CI: 0.53‐0.98; *P*=0.034). |
| Gajewska J. et al., 2019.^21^ | Cohort | 89 children | 5 to 10 y | Poland | *ITLN1*  *rs2274907*  *SERPINA12*  *rs2236242* | PCR-RFLP | BMI  BMI z-score | means ± SD | Children carrying of different genotypes of SERPINA12 rs2236242 had differences in BMI (p =0.025) and BMI Z-score (p = 0.01) values were found. |
| Lee S., 2019.^22^ | Cohort | 692 children  (305 with obesity, 387 without obesity ). | 13.9±0.8 y | Korean | *LRP1B*  rs431809 | Exome sequencing | BMI  WHR | OR, 95% CI | A novel single nucleotide polymorphism (rs431809) in intron 4 was significantly correlated with BMI and WHR. |
| Cardel M. et al., 2019.^23^ | Cohort | 286 children | 7 to 12 y | NonHispanic Black, Hispanic American and NonHispanic White | *TAQ1A*  rs1800497 | Melting Curve | BMI | Mean, SE | Children with two Taq1a risk alleles demonstrated statistically significant higher total body fat, body fat percentage, intra-abdominal adiposity, subcutaneous abdominal adiposity and total abdominal adiposity than did children with one or no Taq1a risk alleles. |
| Ren D. et al., 2019.^24^ | Case-control | 400 adolescents  (222 health controls and 178 with overweight/obesity) | 14 to 18 y | China | *LEPR*  rs1137100  rs8179183  *MC4R*  rs17782313  rs10871777  rs12970134  rs17700144 | MALDI-TOF | BMI | OR, 95% CI | The rs8179183 was significantly associated with the risk of obesity (OR: 3.76, P=0.0004). |
| Ferreira P. et al., 2019.^25^ | Cohort | 871 children and adolescents | 7 to 17 y | Brazil | *IRX3*  rs3751723  FTO  rs9939609 | Allelic discrimination | BMI Z-score | OR, 95% CI | The *IRX3* was associated with the risk of obesity and fat percentage (OR=2.61, P=0.017 and OR=2.24, P=0.007, respectively). An association of *FTO* rs9939609 with BMI Z-score and WC was detected. |
| Yang Y. et al., 2019.^26^ | Case-control | 370 subjects  (170 with overweight and 200 without obesity) | 7 to 18 y | China | *FTO*  rs9939609  rs9935401  *MC4R*  rs12970134  rs17782313 | Allelic discrimination | BMI | OR, 95% CI | The *FTO* rs9939609, rs9935401  and *MC4R* rs1297013 were associated with the risk of obesity (OR=1.79; OR=1.67; and OR=1.52) in the study population. The rs17782313 only showed a trend for obesity risk (P=0.064). |
| Liu H. et al., 2019.^27^ | Cohort | 795 children  (405 without obesity and 390 with obesity). | 5 to 17 y | Mexico | *29 SNPs in MC4R, ETV5*  *TFAP2B*  *LRRN6C*  *PCSK1*  *ETS2, FIGN*  *FOXO3*  *GBE1, GRP*  *HH1P, USP37*  *LOC285762*  *MAF, RPTOR*  *PRKCH* | Fine-mapping SNPs | z-BMI: | OR, 95% CI | Seventeen loci were significantly associated with obesity, and five had fine-mapping SNPs better associated with obesity than their corresponding GWAS index SNPs in Mexican children. |
| Turcotte M. et al., 2019.^28^ | Cohort | 1,421 children and adolescents | 5 to 17 y | Mexican | *12 SNPs in ADAMTS9*  *DNM3/PIGC*  *GRB14* *HOXC13*  *ITPR2/SSPN*  *LYPLAL1*  *NFE2L3*  *NISCH/STAB1*  *RSPO3*  *TBX15/WARS*  *VEGFA*  *ZNRF3/KREMEN1* | TaqMan Open array real-time PCR assay | BMI | β, SE | The only significant association was found between the *GRB14* rs10195252 (β=-0.040, P=3.70×10^−2^) and obesity. |
| Muller Y. et al., 2019.^29^ | Cohort | 1,958 children | 11±3 y | Pima Indian | *TMEM18*  rs2867125  *TCF7L2*  rs7903146  *MRPS33P4* | Allelic discrimination | BMI-z score | β, SD | There was a significant association between BMI z-score with seven variants; the rs2867125 (β=0.131), rs7193144 (β=0.14), rs10938397 (β=0.096), rs1460676 (β=0.143), rs9374842 (β=0.139), rs1441264 (β=0.114) and rs1555543 (β=0.076). |
| Marginean C. et al., 2019.^30^ | Cohort | 188 children  (109 without obesity and 79 with overweight). | 5 to 18 y | Romania | *TNF-α*  308G>A | ARMS-PCR | BMI z-score | OR, 95% CI | A significant association was found between *TNF-a* 308G>A and weight status in the studied population (OR=0.30, P=0.003). |
| Zaharan N. et al., 2018.^31^ | Cohort | 1,151 adolescents | 15 y | 905 Malays  84 Chinese  102 Indians  59 others | *ADRB3*  rs4994  *FABP2*  rs17 | iPLEX GOLD system | BMI | β coefficient, 95% CI | The CC genotype of *ADRB3* rs4994 (β=−0.16) and AA genotype of *MC3R* rs3827103 (β=−0.06) were significantly associated with %body fat compared to TT and GG genotypes, respectively. |
| Dos Santos A. et al., 2018.^32^ | Cohort | 1,211 children and adolescents | 4 to 11 y | Brazil | *LEPR*  rs115650230  rs116239759  rs202069668  rs79353784  rs78005150 | HumanOmni2.5-8 platform using the BeadChip kit | BMI z-score | OR, 95% CI | The SNPs rs115650230 (OR=2.19), rs116239759 (OR=2.25), rs202069668 (OR=1.51) and rs79353784 (OR=2.84; 95%CI: 1.19–6.75) were associated with risk of overweight and obesity.  The rs78005150-G allele was negatively associated (OR = 0.41) with obesity. |
| Col N. et a., 2018.^33^ | Case-control | 200 adolescents  (100 with obesity and  100 without obesity) | 10 to 18 y | Turkey | *MTNR 1B*  rs8192552  rs10830963 | PCR SNaPshot | BMI | OR, 95% CI | No statistically significant association was found between *MTNR1B* gene rs8192552 and rs10830963 with obesity. |
| Almeida et al, 2018.^34^ | Case-control | 773 prepubertal children | NA | Portuguese | *LEPR*  rs1137101 *FTO*  9939609  *MC4R*  rs2229616 rs17782313  *PPARG*  rs1801282 | Allelic discrimination | BMI z-score  BMI | OR, 95% IC | Weak effects of rs17782313 and rs9939609, suggesting a very scarce contribution to childhood obesity. |
| Zandoná, et al., 2017.^35^ | Cohort | 745 children | 3.5 y | Brazilian  55.9% white | *MC4R* rs17782313, *TMEM18* rs6548238,  *BDNF* rs6265 rs10767664 *KCTD15*  rs11084753 *NEGR1* rs2815752 *SH2B1* rs7498665  *SEC16B* rs10913469 *OLFM4* rs9568856  *HOXB5* rs9299. | Allelic discrimination | BMI Z-score,  sum of skinfolds and  waist circumference | ANOVA with adjustment by Benjamini Hochberg with false discovery rate of 0.10. | Significant associations were found at 3.5 years old between TMEM18 rs6548238, NEGR1 rs2815752, BDNF rs10767664 and rs6265 overweight. |
| Dorajoo R et al., 2017.^36^ | Cohort | 1,055 children  (SCORM study) | 9 y | China | 16 SNPs in  FTO, MC4R, TRIM66, TUB, NT5C2, FPGT TNNI3K, FIGN  BCDIN3D, FAIM2, BDNF RABEP1, MIR148A, NFE2L3, GIPR, QPCTL PRKD1, GRP, RASA2, MTCH2  MAP2K5, LBXCOR1 | Illumina550  SNP array | BMI | Weighted gene-risk score (wGRS) | Some common adult BMI risk variants predispose to pediatric obesity risk in East-Asians. The wGRS showed strong association with BMI at age 9, explaining 4.73% of BMI variance. |
| Boyraz M et al., 2016.^37^ | Cohort | 206 participants (143 with obesity and 63 without obesity) | 8 to 16 y | Turkey | *SOCS3*  rs12059  rs1061489  rs17849241  rs2280148  rs8064821  rs12953258  rs4969169 | PCR | BMI | OR, 95% CI. | No association was found between the SNPs in *SOCS3* and obesity. |
| Codoñer-Franch P. et al., 2016.^38^ | Cohort | 180 children | 5 to 17 y | Spain | *RBP4* rs3758538  rs3758539  rs12265684 rs34571439 | Real time PCR | BMI z-score, | OR, 95%CI. | Findings suggest that childhood obesity may be associated with variations in *RBP4* gene. |
|  |  |  |  |  |  |  |  |  |  |
| Reuter C. et al., 2016.^39^ | Cohort | 420 children and adolescents | 7 to 17 y | Brazil | *FTO*  rs9939609 | Allelic discrimination | BMI | OR, CI 95% | Individuals with *FTO* rs9939609-AA showed higher prevalence of overweight or obesity evaluated by BMI (OR: 3.21), waist circumference (OR: 2.59), and % body fat (OR: 2.59). Additionally, the AA genotype had a statistical significant OR for obesity (OR: 4.40 for BMI and OR: 3.54 for waist circumference). |
| Aris I et al., 2016.^40^ | Cohort | 1090 children | 48 months | Singapore | *MC3R* | Illumina omniexpress + exome array | Two skin-folds (triceps and subscapular) | OR, CI 95% | Each additional *MC3R* minor allele copy was also associated with increased overweight (OR=1.48) and obesity (OR=1.58) in the first 48 months. |
| Kaulfers et al., 2015.^41^ | Cohort | 1058 children | 10 to 18 y | African American  Non-Hispanic white (NHW) | *INSIG2*  rs7566605 | SNPlex TM platform | BMI | Coefficient β ± SE | There was no association between overweight and rs7566605, but there was a nominally significant association with overweight and rs17047757 in NHW children (P = 0.043). The G allele of rs17047757 has an OR=1.5 for overweight in children. |
| Shahid A. et al., 2015.^42^ | Cohort | 120 individuals  75 with obesity  45 without obesity | ≤18 y | Pakistan | LEPTIN  G2548A | PCR-RFLP | Children and adolescents ≤18 years of age were divided into obesity (>95^th^ percentile) and no obesity (5^th^ 85^th^ percentile) | OR, CI 95% | A significant association between GA+GG genotype (X^2^=14.6, P=0.0001) was found only in children and adolescents.  According to sex, significant association was found with obesity only in girls (X^2^=14.6, P=0.0001). LEP G-2548A polymorphism increased the risk of obesity in girls (p<0.05). |
| Kilic U. et al., 2015.^43^ | Case -control | 120 children with obesity (70 female and 50 male)  120 children without obesity (78 female and 41 male) | 11.4±3.2 y  12.4±2.1 y | Turkey | SIRT1  rs7895833  rs7069102  rs2273773 | PCR-CTPP | BMI | OR, CI 95% | Carriers of G-rs7895833 had 1.9 times increased odd ratio of having obesity compared with non-carriers (χ^2^: 8.4, p=0.003, OR: 1.9, %95 Cl: 1.2–2.9). Female carriers of G-allele had 0.5 times increased odd ratio of having obesity compared with non-carriers (χ^2^:6.7, p=0.010, OR: 0.48, 95%CI: 0.27–0.84).  For rs7069102 and rs22737773, there were no significant associations. |
| Pillay V. et al., 2015.^44^ | Cohort | 990 adolescents  524 female  466 male | 13.7±0.2 y | South African black adolescent | *GNPDA2*  rs10938397  *MTCH2*  rs10838738  *NEGR1*  rs2568958  *SH2B1*  rs7498665  *STK33*  rs10769908  *TMEM18*  rs6548238 | Illumina VeraCode assay | BMI | OR, CI 95% | Three of the SNPs tested were associated with BMI and showed a consistent directional effect to that observed in non-African cohorts. There was an association between BMI and rs10938397-G (β:0.013) near GNPDA2 (P_adj_ =0.003), rs7498665 (β: 0.007) in SH2B1 (P_adj_ =0.014) and rs6548238 (β:0.011) near TMEM18 (P_adj_ =0.030). This data suggests that common genetic variants potentially contribute to obesity risk in diverse population groups. |
| White M. et al., 2015.^45^ | Cohort | 360 children and adolescent  170 males  190 females | 12.2±0.5 y | Istanbul | SHBG  rs1799941  LPL  rs328  ABCA1  rs1800977  LIPC  rs1800588  CETP  rs708272 | RFLP-PCR | Age- and sex  specific cutoff points of BMI were also used to assess the overweight and obesity status. | OR, CI 95% | The rs1799941 in SHBG was the only variant associated with risk obesity/overweight (OR:3.80, 95%CI: 1.47-9.83, P=0.006) and risk of increased waist circumference (OR:2.67, 95%CI: 1.09-6.49, P=0.031). |
| Rask-Andersen M. et al., 2015.^46^ | Cohort case-control | Swedish cohort  524 children with severe obesity and  524 normal weight  Control:  527 adolescents (without obesity)  Greek cohort | 12.7±3.2 y  9 to 13 y | Stockholm | TMEM18  rs1127484  rs6548238  rs4854344 | SOLiD sequencing platform  TaqMan genotyping assay (replication and validation) | BMI z-score | OR, CI 95% | The rs1127484, rs6548238, rs4854344 were associated with obesity in the case-control cohort of Swedish children and adolescents at the adjusted threshold for significance (OR=0.60, P=1.11x10^-4^; OR=0.66, P=1.33x10^-3^ and OR=0.65 and P=7.79x10^-4^).  In the Greek cohort, the linear regression revealed associations of rs6548238 and rs4854344 with BMI z-score at the adjusted threshold for significance (P < 0·023). The association of rs11127484 did not reach the adjusted level of significance for association with BMI z-score. |
| Todendi P. et al., 2015.^47^ | Cohort | 470 children and adolescents | Children  7 to 9 y  Adolescents  10 to 17y | Brazil | CRP  rs1205  IL-6  rs1800795  rs2069845 | TaqMan assay | BMI and waist circumferen-ce | OR, CI 95% | Carriers of the risk genotype in the rs2069845 gene were associated with risk of developing obesity (OR= 3.07; CI 1.08, 8.72; P=0.03). |
| Wang H. et al., 2015.^48^ | Case-control | 2,969 subjects  850 children with obesity  2,119 children without obesity | 7-17 y | Beijing, China | LIPC  C-514T | Sequenom MassARRAY iPLEX platform | BMI | OR, CI 95% | A significant association between the polymorphism and obesity is observed in boys (OR: 1.34, 95%CI=0.99-1.80, P = 0.042), but not in girls. |
| Fawzy et al., 2015.^49^ | Case-control | 256 subjects  (128 without obesity and 128 with overweight/  obesity) | Control group  11.8±2.9 y  Case group  12.7±3.3 y | Egyptian | ABCA1  rs2230806  rs2066714 | PCR-RFLP | BMI-z score | OR, CI 95% | The rs2230806 increased the susceptibility to obesity (OR: 2.75, 95%CI :1.01–6.12; P=0.014) compared with the control group. This susceptibility could be gender-specific, with higher risk among females. In addition, the A variant was associated with a higher degree of obesity (P=0.001). Individuals with the G variant of rs2066714 showed lower susceptibility to obesity under all genetic models (P<0.05). |
| Hollensted M. et al., 2015.^50^ | Cohort | 1,672 children  (1,022 with overweight/obesity and 650 without obesity) | Case group  11.7±3.2 y  Control group 12.7±3.3 y | Danish | IL6  rs2069845  LEPR  rs1137100  NAMPT  rs3801266  AMD1  rs2796749 | Illumina Infinitum Human CoreExome Bradchip plataform | BMI | OR, CI 95% | The AMD1 rs2796749 was associated with zBMI (β=−0.16, P=0.0077) while IL6 rs2069845 was modestly associated with zHeight (β=−0.05, p=0.047). |
| Hardman et al., 2014.^51^ | Longitudinal | 3,720 children | 7, 8, 9,10 and 11 y | European | DRD2 rs1800497  OPRM1  rs1799971  FTO  rs1558902 | Illumina HumanHap  550  quad genome-wide SNP genotyping platform | BMI and waist circumference at 5 time points. | Analysis of genotype-by-time interaction.  Regression analysis to predict the per  high-risk allele effect on phenotypes. | DRD2 and OPRM1 genotypes were not associated with adiposity. |
| Zhu W. et al., 2014.^52^ | Case–control | 763 children (194 without obesity and 569 with obesity or overweight) | Control  10.9±2.7 y  Obesity/ overweight  10.7±2 y | Chinese | APOA5  rs662799  rs651821 | Matrix-Assisted  Laser Desorption/Ionization Time of Flight Mass Spectrometry | BMI | Logistic regression analysis was performed  adjusted for age and sex | Genetic variants in the *APOA5* gene may influence the susceptibility of the individual to obesity |
| Albuquerque D. et al., 2014.^53^ | Cross-sectional | 730 children  (256 normal weight, 320 with overweight and 154 with obesity) | 6-12 y | Portuguese | PPARGC1A  rs8192678  MSRA  rs545854  TFAP2B  MC4R  rs987237  rs17782313  rs12970134  NRXN3  rs10146997  TMEM18  rs7561317  SEC16B  rs10913469  HOXB5  rs9299  OLFM4  rs9568856 | Allelic discrimination assays | BMI, BMI Z-score, and waist circumference | Logistic regression under an additive genetic mode adjusted  for age and sex, by calculating odds ratios (ORs) with 95% of CI | TFAP2B rs987237 and MC4R rs12970134 polymorphisms showed an  opposite direction of effect to that in the original reports |
| Marcovecchio M. et al., 2014.^54^ | Case- control | 745 prepubertal children | 6–12 y | Caucasian  Italy | Nineteen SNPs in 17 genes including *DNM3, HHEX LYPLAL1*  *TBX15*  *GRB14*  *TMEM18*  *IRS1,CDKAL1 CDKAL1 RSP03 TFAP2B SLC30A8 CDKN2B, HOXC13, IRS2, FTO MAF, MC4R* | MassARRAY iPLEX | No obesity  (BMI <85^th^ percentile)  With overweight  (BMI 85–95^th^ percentile)  With obesity  (BMI >95^th^ percentile) | Logistic and linear regression analyses using an additive genetic model, adjustments for age, sex and puberty were made when required. | The rs12970134 MC4R variant was significantly associated with excess body weight, particularly in  children older than 8 years of age. |
| Oana M. et al., 2014.^55^ | Case-control | 222 children  (110 without obesity and  102 with obesity) | 1 to18y | Caucasian  Romania | *IL-6*  572C/G  190C/T  174 G/C | PCR-RFLP | BMI for age above the 95^th^ percentile according  to World Health Organization standard | OR, 95%CI | IL-6 572 CC, IL6 190 CC, and IL-6 174 CG genotypes appeared more frequently in children with obesity. |
| Meng X. et al., 2014.^56^ | Case-control | 2,030 children from  ALIR and  CPOOA  cohorts | Control group  14.8±0.7 y  Case group  14.6±0.6 y | Chinese | PP13439-TMEM212  rs6794092  ROPN1L  rs2967951  CDH12  rs268972  MFAP3-GALNT10  rs2033195  MFAP3-GALNT10  rs815611  FTO  rs62048402  rs9939609  FER1L4  rs6088887 | MassARRAY  System  Tetra-primer amplification refractory mutation system analysis | BMI | OR and 95%CI | rs9939609 and rs62048402) with BMI or obesity reached nominal significance at P<0.05. A  cumulative effect of five SNPs on the risk of overweight and  obesity (OR=1.197, 95%CI  =1.06–1.34, P=0.002) was found. Subjects carrying 9 or more effect  alleles had a 127% increased risk of overweight and obesity.  (OR = 2.2, 95%CI=1.40–3.67, P=0.001) compared with  subjects who carry 6 or fewer effect alleles |
| Muller Y. et al., 2014.^57^ | Longitudinal study | 5,350 children and adolescents | 13.8±3.9 y | Pima  American Indians | 8 tag SNPs and rs17782313 in the MC4R region | TaqMan Open Array System  And BeadXpress | BMI z-score | Linear regression using the generalized estimating equation procedure. | Association between rs11872992 and childhood BMI z-score (β=0.11, p=0.01). One haplotype of the G allele at rs74861148 and the A allele at rs483125 provided the strongest association with childhood BMI z score (β=0.08, p=0.001) |
| Ng Z. et al., 2014.^58^ | Case-control | 613 adolescents | 14.8±1.3 y | 241 Malay,  219 Chinese,  153 Indian | LEP rs7799039  LEPR  rs1137101  TNF-α  rs1800629 | PCR-RFLP | BMI  BMI z-score  Fat % | OR, 95% CI | LEP G-2548A allele may be associated with overweight/obesity in Indian  male adolescents in Malaysia. |
| Ruan L. et al., 2014.^59^ | Case–control | 400 adolescents with obesity and 200 adolescents without obesity | 10.5 ±2.0 y | Chinese | *HSD11B1* rs4393158 rs2235543 rs10082248  rs10863782 rs2236903 rs2298930 rs4545339 *HSD11B2* rs28934592 rs28934591  rs28934594 rs28934593 | MassArray | Obesity  BMI Z score  BMI | Logistic regression analysis in five SNPs under different genetic models with OR, 95%CI | *HSD11B1* may be a  cause of childhood obesity, or even associated with the  complication of childhood obesity |
| Shinozaki K. et al., 2014.^60^ | Longitudinal | 66 children | 3, 10 and 13 y | Japanese | *FTO*  rs1558902 | Allelic discrimination | Overweight in children was defined using the IOTF cutoffs. | General linear models adjusting for sex and age | The increment of adiposity at 10 years of age in boys might be influenced  by the *FTO* variant, but this influence was significantly reduced at 13 years. |
| Wu J. et al., 2014.^61^ | Case-control | 401 adolescents  223 without obesity, 178 with overweight | 16.3±1.5 y | Chinese | *FTO*  rs9939609  rs1558902 rs8050136 rs6499640  rs3751812 | Sequenom iPlex  MassARRAY | Age and sex specific BMI cut-offs provided by Working Group of Obesity in China (WGOC) | OR, 95%CI | No significant association was found between *FTO* variants and the analyzed phenotypes. |
| Xu et al., 2014.^62^ | Case-control | 500 children with obesity  500 matched children without obesity | Case group  11.8±2.8 y  Control group  11.8±2.9 y | Chinese | 24 SNPs in *FTO* | SNPScan | BMI | Multivariate logistic or linear regression was  Used, adjusted for potential confounders  such as age, gender and location.  Genetic risk score | A dose-response relationship was found between the number of risk alleles in *FTO* rs7206790, rs11644943 and rs9939609 and the risk of obesity. |
| Yang et al., 2014.^63^ | Cros-sectional study | 1,400 adolescents with obesity  2,600 adolescents without obesity | 11.0±2.6 y | Chinese | *FTO*  rs9939609 | Allelic discrimination | Obesity was  defined as  BMI > 95^th^ percentile of the Chinese BMI  reference data for Han children and adolescents in specific age and gender. | OR, 95%CI | *FTO* rs9939609 was significantly associated with the risk of obesity |
| Zhang et al., 2014.^64^ | Cros-sectional study | 757 children with obesity and 2,746 without obesity | 6 to 18 y | Chinese | *FTO* rs9939609 | allele-specific real-time polymerase chain reaction (RT-PCR) | age- and sex-specific  BMI cut points recommended by the International Obesity Task  Force (IOTF) | OR, 95%CI | The association between *FTO* rs9939609 and obesity-related traits may change from childhood to adolescence  in Chinese individuals |
| Ho-Urriola J. et al., 2014.^65^ | Case-Control | 139 children without obesity  238 children with obesity | 6 to 12 y | Chile | *MC4R*  rs17782313 | TaqMan Assay | Obesity were defined as those above the 95^th^ percentile. | OR, CI 95% | The *MC4R*-rs17782313 C carriers did not achieve statistical significance with obesity (OR=1.4; 95% CI= 0.8-2.4; P=0.16). |
| Albuquerque D. et al., 2013.^66^ | Cohort | 730 children  256 without obesity and  320 with overweight  154 with obesity | 6 to 12 y | Portugal | *FTO*  rs9939609  rs1421085  rs1861868 | TaqMan assay | Overweight was classified by BMI cut-points between ≥25 kg/m^2^ and <30 kg/m^2^ and obesity ≥ 30 kg/m^2^. | OR, CI 95% | There were significant associations of rs9939609 and rs1421085 with weight, BMI, BMI-z, circumference and hip circumference (p<0.05 in all traits). rs1861868 was marginally associated with weight (p=0.081) and BMI (p=0.096). In case-control studies, both rs9939609 and rs1421085 were significantly associated with obesity (OR=1.97; p=0.026; OR=2.11; p=0.013, respectively) but not with overweight (p>0.05). |
| Chaves et al., 2013.^67^ | Case control | 120 adolescents with obesity  150 adolescents without obesity | 12.8±0.1 y  13.7±0.2 y | Euro-Brazil | *BCHE*  rs1126680  rs1803274 | TaqMan assay | The BMI was standardized to BMI z-score. | X^2^-test | These results shown a protective effect of -116A and K variants on juvenile obesity risk. |
| Chen Y. et al., 2013.^68^ | Cohort | Chinese children cohort:  142 children with severe obesity  384 children without obesity  Longitudinal  Chinese cohort from Da Qing without Obesity | 11.6 y | China | *NUCB2*  rs757081 | TaqMan assay | The percentage of ideal weight for height (WFH) was used as an acceptable measure of degree of childhood adiposity. | OR, CI95% | CC/CG carriers had a significant association with obesity (OR=2.29, P=0.013) in the dominant model, CC (OR=2.86, P=0.007) in the additive model, and C allele (OR=1.57, P=0.002). In the replication cohort there were an association with obesity in CC and CG carriers (OR=1.69). Within the Da Qing cohort, subjects with the GG genotype had significantly lower BMI and percentage ideal weight for height at 5 and 8 years of age. It is postulated that GG genotype is protective against excessive weight gain. |
| Rupérez A. et al., 2013.^69^ | Case-control | 189 children  179 children with obesity | 4 to 13 y | Spanish | *PON1*  rs13236941  rs757158  rs705382  rs854573  rs854572  rs854571  rs854566  rs705378  rs854560  rs3917527  rs662  rs854552  rs854551  rs3735590  rs854550 | Illumina Colden Gate Assay | Obesity was defined according to BMI, using the age- and sex-specific cut-off points proposed by Cole et al. | OR, CI 95% | Among the sixteen genotyped *PON1* SNP, only the intronic SNP rs854566 exhibited a significant association with obesity (OR=0.61, 95% CI= 0.41-0·91; P=0.016). |
| Wang L. et al., 2013.^70^ | Case-control | 158 children with obesity  397 children without obesity | 3 to 6 y | China | FTO  rs1421085  rs17817449 | PCR-restriction fragment length polymorphism (RFLP) assay. | Weight-for-height was considered as a useful index to define the status of weight in preschool children (WHO 1995). Children with >120% were diagnosed with obesity. | OR, CI 95% | The *FTO* -CT/CC genotypes were associated with 59% and 71% increased risks of childhood obesity (OR=1.59, 95%CI=1.00-2.53 for CC; OR=1.71, 95%CI =1.10-2.65 for CT/CC) compared with wide-type. The rs17817449 T > G variant was not significantly associated with the risk of childhood obesity. |
| Rupérez A. et al., 2013.^71^ | Case-control | 194 children  191 children with normal weight | 3 to 13 y | Caucasian (Spain) | *CAT*  rs564250  rs769214  rs7943316  rs1049982  rs525938  rs494024  rs769218 rs12269988 rs2073058 rs4755374 rs12803540 rs7104301 | Illumina GoldenGate (Illumina) array | Obesity was defined according to BMI, using the age- and sex-specific cut-off points proposed by Cole et al. (linked to adult cut-offs of 25 and 30 kg/m2). | OR, CI 95% | Only the rs769214, rs7943316 and rs1049982 in *CAT* gene were associated negatively with childhood obesity (OR=1.42, p=0.035; OR=1.38, p=0.050 and OR=1.41, p=0.039, respectively). |
| Huen K. et al., 2013.^72^ | Cohort | 386 children (at 2 y)  331 children (at 5 y) | 2 and 5y | Mexican-American (California) | *PON1*  PON1_192_ | TaqMan Assay | Children with a BMI at or above the 95th percentile of the 2000 CDC sex-specific BMI- for-age growth charts were considered with obesity. | OR, CI 95% | A significantly higher OR of obesity (9.3 and 2.5- fold) was found in PON1192_QQ_ children compared to PON1192_RR_ at ages two and five, respectively. Similar relationships were seen with BMI Z-scores at age two and waist circumference at age five. After adjusting for genetic ancestry in models of *PON1* and BMI Z-score, effect estimates for PON1192 genotype changed 15% and 9% among two- and five-year-old children, respectively. |
| León-Mimila P. et al., 2013.^73^ | Cohort | 1,218 children  595 boys  658 girls | 6 to 15 y | Mexican mestizos | *ADIPOQ*  rs2241766  *UCP3*  rs1800849  *FTO*  rs9939609  *TMEM18*  rs6548238  *INSIG2*  rs7566605  *FAIM2/BCDN3*  rs7138803  *BDNF,* rs6265  *GNPDA2*  rs10938397  *SH2B1*  rs7498665  *MC4R*  rs17782313  *KCTD15*  rs29941  *SEC16B/RASAL2*  rs10913469 | TaqMan Assay | BMI z-scores were calculated using age and sex specific BMI reference data, as recommended by the Centers for Disease Control and Prevention. | OR, CI 95% | The rs7138803 and rs10938397 SNPs were significantly associated with BMI (β=0.33, SE: 0.13, P=0.008 and β=0.09, SE: 0.04, P=0.021, respectively), while the rs7138803 was associated with WC (β=0.28, SE: 0.12, P=0.024). |
| Olza J. et al., 2013.^74^ | Case-control | 292 children with obesity  242 children without obesity | 5 to 15 y | European-Caucasian (Spain) | *NPY*  rs16147  rs16478  rs16135  rs16131 | Golden Gate array (Illumina). | Obesity was defined according to the International Obesity Task Force (IOTF) reference for children. | β, SE | Only the rs16147 and rs16131 SNPs were significantly associated with risk of obesity (OR=1.38, p=0.045 and OR=1.69, p=0.016, respectively) in the cohort study. The rs16147 was associated with weight, BMI and BMI z-score (p<0.05). |
| Goumidi L. et al., 2013.^75^ | Cohort | 1,155 adolescents from HELENA Cohort | 12 to 18 y | European | *REV-ERBα*  rs2071427 | TaqMan assay | Overweight was defined according to Cole et al.  who reported international cut off points for BMI by age and sex, corresponding to at least 25 kg m^-2^  at the age of 18. | OR, CI 95% | There was a significant association between the T-rs2071427 and higher BMI (p=0.003) and overweight (OR=1.49, 95%CI: 1.09-2.04, P=0.001). |
| Wheeler E. et al., 2013.^76^ | Cohort | 1,509 children with extreme obesity  5,380 children without obesity | --- | Caucasian | *FTO*  rs1421085  *MC4R*  rs476828  *TMEM18*  rs12463617  *NEGR1*  rs1993709  *PRKCH*  rs1957894  *LEPR*  rs11208659  *PACS1*  rs564343  *RMST*  rs11109072  *NEGRI*  rs3101336 | TaqMan assay | Extreme obesity was defined according to the early-onset obesity was defined according to BMI standard deviation score (SDS)>3. | OR, CI 95% | There were significant risk alleles associated with severe, early-onset obesity in the children cohort rs1421085 (OR=1.49), rs476828 (OR=1.33), rs12463617 (OR=1.43), rs1993709 (OR=1.46), rs1957894 (OR=1.64), rs11208659 (OR=1.63), rs564343 (OR=1.25), rs11109072 (OR=1.79) and rs3101336 (OR=1.26), (P<0.05 all of them). |
| De la Cruz-Mosso U. et al., 2013.^77^ | Cohort | 174 children  89 without obesity  85 with obesity | 6 to 13 y | Mexican | *PAI-1*  -675 4G/5G | PCR-RFLP | Obesity was defined as ≥ 95^th^ percentile. | β, CI95% | The 4G/5G genotype contributed to a significant increase in waist-hip ratio (β=0.02, p=0.006), waist circumference (β=4.42, p = 0.009), and subscapular skinfold (β=1.79, p=0.04). |
| Ntalla I. et al., 2013.^78^ | Cohort | 707 adolescents | 13.4±0.8 y | Greek | *FTO*  rs1558902  *TMEM18*  rs2867125  *FAIM2*  rs7138803  *RBJ*  rs713586  *QPCTL*  rs2287019  *ZNF608*  rs4836133 | Genotypes were imputed using data from the HapMap II genotype data from the 60 CEU HapMap founders. | Obesity, overweight and normal weight were defined according to the age- and sex-specific criteria by International Obesity Task Force (IOTF) | β, SE  OR, CI95% | Variants at the *FTO, TMEM18, FAIM2, RBJ, ZNF608* and *QPCT*L loci yielded nominal evidence for association with BMI and/or overweight risk (p<0.05). Variants at *TFAP2B* and *NEGR1* loci showed nominal association (p<0.05) with BMI and/or overweight risk in males and females respectively. |
| Rask-Andersen M. et al., 2013.^79^ | Case-control cohort  Cross-sectional cohort | 991 children and adolescents  (518 without obesity and  473 with obesity)  2,308 children | 17±0.9 y  12.8±3.2 y  11.2±0.7 y | Swedish  Greek | *STK33*  rs4929949 | TaqMan assay | BMI-z-score was calculated according to the Obesity Task Force (IOTF) definitions. | OR, CI 95% | The minor allele of rs4929949 was associated with obesity in the cohort of Swedish children and adolescents (OR=1.199, p=0.047), and with body mass in the cross-sectional cohort of Greek children (β=0.08147, p=0.021). |
| Mejía-Benítez A. et al., 2013.^80^ | Cohort | 1,463 children  514 cases  949 controls | 6 to 12 y | Mexican | *FTO*  rs1421085  *NPC1*  rs1805081  *ENPP1*  rs7754561  *NEGR1*  rs2815752  *GNPDA2*  rs10938397  *MC4R*  rs17782313 | TaqMan assay | Overweight was defined as a BMI-for-age between the 85^th^ and 95^th^ percentiles, while obesity was defined when BMI-for-age was higher than the 95^th^ percentile in WHO charts. | OR, CI 95% | There was a significant effect of GNPDA2 rs10938397 on risk of obesity (OR=1.30; P=1.34 ×10^-3^). There were a nominal association between obesity risk or BMI variation and the following SNPs:  ENPP1 rs7754561 (OR=0.84; P=0.020) and  MC4R rs17782313 (OR=1.40; P=0.016).  NEGR1 rs2815752, NPC1 rs1805081 and FTO rs1421085 did not reach significant association. |
| Xi B. et al., 2013.^81^ | Cross-sectional study | 3,502 children and adolescents  1,196 children with obesity  2,306 children without obesity | 6 to 18 y | Chinese | *FTO*  rs9939609  *MC4R*  rs17782313  *GNPDA2*  rs10938397  *BDNF*, rs6265  *FAIM2*  rs7138803  *NPC1*  rs1805081  *SEC16B*  rs10913469  *SH2B1*  rs4788102  *PCSK1*,  rs6235  *KCTD15*  rs29941  *BAT2*  rs2844479 | TaqMan assay | The 90th percentile values of WC for age and sex and WHtR cut- off of 0.5 to diagnose children with central obesity. | OR, CI 95% | Of 11 SNPs analyzed, four SNPs were statistically significantly associated with central obesity by WC criteria (*FTO* rs9939609: OR=1.29, p=0.001; *MC4R* rs17782313: OR=1.27, p=1.3x10-^4^; *GNPDA2* rs10938397: OR=1.22, p=4.09x10^-4^; *BDNF* rs6265: OR=1.20, p=8.86x10^-4^) after adjustment for sex, age, pubertal stage, physical activity and family history of obesity. Similar observations were made using weight-to-height ratio (WHtR) criterion. However, other SNPs were not associated with central obesity by WC as well as WHtR criterion. |
| Xi B. et al., 2013.^82^ | Cohort | 2,849 children and adolescents  1,230 children with obesity  1,619 without obesity | 6 to 18 y | Chinese | *SEC16B*  rs10913469  *SH2B1*  rs4788102  *PCSK1*  rs6235  *KCTD15*  rs29941  *BAT2*  rs2844479 | TaqMan assay | WC and waist-to-height ratio (WHtR, equal to WC/height) were used as measures of central adiposity. | OR, CI 95% | Of five studied variants, only two variants *SEC16B* rs10913469 and *SH2B*1 rs4788102) were nominally associated with the risk of obesity in the total population (OR=1.19, p=0.012 and OR=1.17, p=0.041), while no statistical associations were found for three other variants. |
| Olza J. et al., 2013.^83^ | Case-control | 534 children  292 children with obesity  242 children without obesity or overweight | 6 to 15 y | Caucasian (Spain) | *FTO*  rs9935401  rs9939609  rs9928094  rs9930333 | Illumina GoldenGate Assay. | Obesity was classified according to BMI, using the age- and sex-specific cut-off points of Cole et al. | OR, CI 95% | Four polymorphisms rs9935401 (OR=1.76, p=0.004), rs9939609 OR=1.73, p=0.009), rs9928094 OR=1.66, p=0.018) and rs9930333 OR=1.64, p=0.023) were associated with the risk of obesity.  The rs8061518 was negatively associated with obesity (OR=0.56, p=0.025). |
|  |  |  |  |  |  |  |  |  |  |
| Sallman M. et al, 2013.^84^ | Cohort | 524 children with severe obesity  527 adolescents without obesity | 12.7 y  17.0 y | Swedish | *FTO*  rs9930333  rs9930397  rs9923544  rs11642015  rs1558901  rs62048402  rs11075985  rs17817288  rs55872725  rs1121980  rs7193144  rs9941349  rs28567725  rs9930501  rs9930506  rs9932754  rs9922708  rs9922619  rs11860076  rs11864972 | GoldenGate and TaqMan | Not specified | OR, CI 95% | Ten obesity-associated SNPs had a stronger obesity association such as rs9930333 (OR=1.29), rs9923544 (OR=1.28), rs11642015 (OR=1.33), rs62048402 (OR=1.35), rs11075985 (OR=1.29), rs17817288 (OR=0.79), rs55872725 (OR=1.33), rs1121980 (OR=1.27), rs9941349 (OR=1.30), rs28567725 (OR=1.29)  (P<0.007) than the commonly studied rs9939609 SNP (P<0.012). |
| Csemus K. et al., 2013.^85^ | Cohort | 1,346 children  (709 with  Overweight/ obesity and  637 without obesity) | 6 to 17 y | Hungary | *BAR*  Trp64Arg  *UCP-1*  -3826 A/G  *UCP-2*  Exon 8 del/ins  *UCP-2*  -866 G/A  *UCP3*  -55 C/T  *PPARG*  Pro12Ala | PCR/PCR- RFLP method | Overweight or obesity was defined according to International Task Force cut-off values for BMI by sex and age. | OR, CI 95% | On multivariate regression analysis adjusted for age and gender heterozygosity and homozygosity for the *UCP-*2 –866, a variant was associated with obesity (OR=0.69; p=0.013 and OR=0.50,  p=0.003, respectively) compared with G/G. Heterozygotes and homozygotes for the *UCP-*2 exon 8 allele had an OR=1.66, p=0.001 and OR=2.12; p=0.006, respectively. There were no significant differences in obesity risk in association with the other examined gene polymorphisms. |
| Albuquerque D. et al., 2013.^86^ | Cohort | 580 children  (140 with obesity, 233 with overweight and 207 without obesity) | 6 to 12 y | Portugal | *LCT*  -13910 C>T | TaqMan assay | Obesity was defined according to the IOTF cut-offs and abdominal obesity using the sex and age-specific ≥ 90^th^ waist circumference percentile. | OR, CI 95% | There were association between the-13910*T allele and children abdominal obesity (OR=1.41; p=0.030). Under the dominant model, the indicative association was observed between the LCT-13910 CT/TT genotypes and abdominal obesity, remaining significant after adjustment for age and gender (OR=1.65; p=0.029). No association was detected with the risk of obesity (p=0.350). |
| Dwivedi O. et al., 2013.^87^ | Cohort | 1,362 children (927 with normal weight, 435 with overweight and 159 with obesity) | 11 to 17 y | India | *MC4R*  rs17782313  rs12970134 | iPLEX assay | Obesity was classified using age- and sex-specific BMI cutoffs provided by Cole et al. | OR, CI 95%  β, 95% CI | Both rs17782313 and rs12970134 SNPs showed strong association with adiposity measures (weight, body mass index and waist circumference) in children (P_range_=7.6x10^–5^ - 3.8x10^–12^). |
| Chen Y. et al., 2013.^88^ | Cohort | 526 children (142 with severe obesity and  384 without obesity)  762 children from replication cohort  (372 with obesity and  390 without obesity) | 5 to 10 y  6 to 9 y | China | *NUCB2*  rs757081 | TaqMan assay | BMI and adiposity were defined according to  School Health Service, Ministry of Health, Singapore. | OR, CI 95% | CC/CG carriers had a significant risk for obesity (OR=2.29) in the dominant model.The CC genotype also had a significant association with obesity (OR=2.86, 95%). The carriers of the C allele showed a risk association with overweight (OR=1.57). These findings were replicated in an independent cohort of Chinese children (OR=1.69). All associations had a p<0.05. |
| De la Cruz-Mosso U. et al., 2012.^89^ | Case-control | 100 children | 6 to 11 y | Mexican mestizo | *PAI-1*  -844G/A  Hindlll C/G | PCR-RFLP | Obesity was defined according to the obesity ≥ 95th percentile. |  |  |
| Jacobsson J. et al., 2012.^90^ | Case-control study | 1,076 children  2,249 children | 11.2 y | Swedish | *MGAT1*  rs12186500  rs1021001  rs4285184 | Allelic discrimination, TaqMan assay | Overweight and obesity were defined using International Obesity Task Force BMI cut-off values equivalent to adult BMI of 25 and 30, respectively. |  |  |
| Hsieh C. et al., 2012.^91^ | Cohort | 925 adolescents  451 boys  474 girls | 11 to 15 y | Taiwan | *IL-6R*  rs8192284 | TaqMan assay | Obesity is defined as the 90^th^ percentile BMI or waist circumference according to age and gender specifications. | OR, CI 95% | The OR for a high WC C-allele carriers were  OR=1.54 (95% CI 1.01-2.34) when compared with A-allele carriers. |
| Wang J et al., 2012.^92^ | Association study | 1,688 children | 10 to 12 y | China | Eight SNPs within the *NEGR1*  *TMEM18*  *GNPDA2*  *MTCH2*  *SH2B1*, *FTO* *MC4R*  *KCTD15* | TaqMan assay | Weight, BMI, waist-to-height ratio and body fat | OR, CI 95% | Variants of *TMEM18* and FTO were associated with obesity indices during puberty in Chinese children. rs9939609 showed a strong association only in girls. |
| Durán-Gonzalez J. et al., 2011.^93^ | Case-control study. | 448 adolescents | 16 ±3.5 y | Mexican Americans | *HSD11B1*  rs846910  *CRP*  rs1205  *ADIPOQ*  rs1501299  *PPARG*  rs1801282  *ANKK1*  rs1800497  *ABCC8*  rs757110  *SERPINF1*  rs1136287 | Allelic discrimination | BMI, WC, obesity, non-obesity | OR and 95% IC | The SNP rs1800497 (*ANKK1*) had association with WC. No association between rs846910 (*HSD11B1*), rs1205 (*CRP*),  rs1501299 (*ADIPOQ*), rs1801282 (*PPARG*), rs757110 (*ABCC8*), and rs1136287  (*SERPINF1*) and obesity was found. |
| Gao L. et al., 2011.^94^ | Case-control study. | 615 children  (409 with obesity/overweight and  206 without obesity) | 13*.*4±2*.*3 y | Chinese | *GHR exon 3 d3-GHR* | PCR | BMI | --- | The d3-GHR polymorphism has a significant effect on BMI and metabolic parameters of Chinese children with obesity. The d3 allele may have a protective effect on the development of metabolic syndrome by increasing insulin sensitivity. |
| Horvatovich K. et al., 2011.^95^ | Case-control | 369 children  (232 with obesity and 137 without obesity) | 13.7±0.1 y | Hungarian | *APOA5*  T-1131C  IVS3  G476A  1259C  T1259C C56G | PCR/RFLP | BMI | OR, 95% CI | *APOA5* *5 haplotype (1259C alone) can be protective against obesity. |
| Bokor S. et al., 2011.^96^ | Case-control and cross- sectional | 544 adolescents (214 with obesity and 330 without obesity)  1,155 adolescents | 14.7 y | European | 53 SNPs in genes: *MTHF. MTR, MTRR, CBS,* *TCN2* and *PON1*. | Illumina system | BMI, percentage body fat and waist circumference. | OR, 95% IC | There was no evidence for any association between SNPs of *MTHFR*, *MTR*, *CBS*, *TCN2* and *PON1.* Two SNPs of *MTRR* were associated with a higher (rs10520873, OR: 1.68) or lower (rs1801394, OR:0.61) risk of obesity. In the cross-sectional sample, rs1801394 was associated with lower BMI and lower waist circumference. |
| Lin F. et al., 2010.^97^ | Cohort | 893 children | 13.1 y | Taipei | *CRP*  +2147  IL-6R rs2229238 | Allelic discrimination | BW, BMI, WC, HC, BF% and WHtR. | OR, 95% CI | Boys who carry the GG genotype of *CRP* +2147 A/G and the CC genotype of IL-6R rs2229238 C/T had a greater risk of having higher BW, BMI, WC, HC, BF and WHtR and of developing obesity compared individuals who did not have these genotypes. |
| Zhu J. et al., 2010.^98^ | Case-control | 330 children and adolescents  (230 with obesity and 100 without obesity). | 6 to 17 y | China | *GHRELIN*  Arg51Gln  Leu72Met  Gln90Leu | PCR-RFLP | BMI | Chi-square (X^2^) and mean ± SD | The Leu72Met polymorphism of *GHRELIN* gene was not associated with obesity in Chinese children. |
| Bollepalli S. et al., 2010.^99^ | Cohort | 1,058 children and adolescents | 10 to 18 y | African Americans | *FTO*  47 SNPs | SNPlex Genotyping System | Weight, waist circumference, BMI and BMI-z | Weight, Eaist circumference, BMI, BMI z score | 17 SNPs were associated with BMI *Z*-score in the study population, the strongest association was found in the African Americans. The most significant SNP was rs8057044, which was associated with BMI-*Z* in the African Americans. The rs9939609 SNP was significant in the non-Hispanic white population. |
| Solaas K. et al., 2010.^100^ | Cross-sectional study | 1,144 adolescents from HELENA study | 14.8±1.4 y | Europe | Twenty SNPs in *LXRβ* | Illumina system using GoldenGate technology. | BMI | OR, 95% CI | It was identified significant associations between rs17373080 and overweight/obesity in HELENA cohort (OR=1.59, p=0.002). |
| Sauber J. et al., 2010.^101^ | Cohort | 2,280 children and adolescents  (1,696 normal weight and 584 with obesity | 2 to 18 y | German | *GIPR*  *rs8111428*  *rs2302382*  *rs1800437* | MALDI-TOF | BMI SDS above 2: homeostatic model | OR, 95% CI | No evidence for an association was found between the SNPs and the obesity phenotype. |
| Muller T. et al., 2010.^102^ | Cohort | Trios cohort: 521 children with obesity Families cohort: 92 children with extreme obesity | Trios cohort: 13.4±3.1 y  Families cohort: 13.4±2.6 y | German | *FAAH* rs324420 rs324419  rs873978  rs2295632 rs932816 | MALDI-TOF  PCR-RFLP  Allelic discrimination | BMI | OR, 95% CI | The trio analysis revealed association of *FAAH-*rs324420, rs324419 and rs873978 with childhood obesity. Although these variants in 235 obesity families did not result in significant effects, the combined analysis in 603 obesity families supported the association of *FAAH-*rs324420 and rs2295632 with early onset extreme obesity. |
| Wu L. et al., 2010.^103^ | Cohort | 3,503 children  (1,229 with obesity, 655 overweight and 1,619 without obesity). | 6 to 18 y | China | *FAIM2*  rs7138803 *NPC1*  rs1805081 *FTO*  rs6499640 *MC4R*  rs17782313  *BDNF*, rs6265  *GNPDA2*  rs10938397 | Allelic discrimination | BMI and obesity | OR, 95% CI | The SNPs rs17782313, rs6265, and rs10938397 were associated with an increase of BMI and obesity in Chinese children. The SNPs rs17782313 and rs10938397 were associated with an in waist circumference, waist-to-height ratio, and fat mass. |
| Ma L. et al., 2010.^104^ | Case-control | French children (685 control and 707 case)  German children (715 control and 283 case) | 10 to 12 y | French  Germany | *A2BP1*  rs10500331 rs12924838 | Illumina Human CNV370 Duo Array and allelic discrimination | BMI | Linear regression models | No single variant in *A2BP1* was associated with BMI or obesity in French and German children. |
| Riestra P. et al., 2010.^105^ | Cohort | 806 children | 12 to 16 y | Spain | *LEPR*  Q223R  K109R  K656N | Allelic discrimination | Body weight, BMI, Hip circumference, waist circumference | X^2^ | Female carriers of Q223R-RR had significantly higher BMI (22.5 vs 21.3 Kg⁄m^2^) than QR carriers. The RR-frequency in overweight-obese girls was higher than normal-weight girls. No differences were found in boys. |
| Xi B. et al., 2010.^106^ | Cross-sectional | 3,503 children | 6 to 18 y | China | *FTO*  *rs9939609* | RT-PCR | BMI, body weight, waist circumference | OR, 95% CI | A significant association between rs9939609 with weight, BMI, waist circumference, waist-to-height ratio, and %fat mass in children and adolescents were found. Each additional copy of the rs9939609-A allele was associated with a BMI increase of 0.79 kg/m^2^. |
| Vimaleswaran K. et al., 2010.^107^ | Cohort | 1,224 children and 838 adolescents | Children 8.4-11.3y  Adolescents 14–17 y | Denmark  Estonia  Norway  Portugal | *PCK1* rs1328756  rs6070157  rs5707555  rs2070755  rs2179706  rs1042531  rs28359554 | Illumina BeadStation custom array | BMI, waist circumference and skinfolds | Mean, SE. | None of the polymorphisms were significantly associated with BMI, waist circumference, skinfolds, or with the risk of having overweight or obesity(*P*>0.05). |
| Pyrzak B. et al., 2010.^108^ | Case-control | 180 participants (124 with obesity and 56 without obesity) | Obesity 10-18 y  Control  11-18 y | Poland | *TNF-α*  G-308A | PCR–RFLP | BMI | OR, 95% CI | Carriers of A (AG+AA) allele among children with obesity were significantly more frequent than in the control group (OR = 2.29, P<0.05). |
| Mo J. et al. 2010.^109^ | Cohort | 265 children (147 with obesity and 118 without obesity) | Not specified | Chinese | *PTP1B*  IVS6+G82A Pro303Pro | PCR-RFL | Waist circumference, waist to hip ratio, percentage of body fat | OR, 95% CI | *PTP1B* gene Pro303Pro polymorphism might be associated with the pathogenesis of obesity in children. |
| Ruiz J. et al., 2010.^110^ | Cross-sectional study. | 752 adolescents | 13 to15 y | Europe | *FTO*  rs9939609 | GoldenGate technology | Weight, waist circumference, skinfolds, BMI and body fat percentage. | Mean, SD  β coefficient, 95% CI | The *FTO*-A allele was significantly associated with higher BMI (+0.42 per risk allele), higher %body fat (+1.03% per risk allele), and higher waist circumference (+0.85 cm per risk allele). |
| Almén M. et al., 2010.^111^ | Cross-sectional study | 1,027 participants (502 children with obesity and 525 without obesity) | Obesity group:  12.6±3.3 y  Control group:  17.1±0.8 y | Swedish | *TMEM18*  rs6548238 rs7561317 | Allelic discrimination | BMI | OR, 95%CI | There was a strong association for two SNPs (rs6548238 and rs756131) of the *TMEM18* gene with an increased risk for obesity. |
| Fang H. et al., 2010.^112^ | Cohort | 670 children | 9.3±0.8 y | China | *FTO*  rs9939609 | Allelic discrimination | BMI, skinfolds | OR, 95% CI | The rs9939609 variant was associated with obesity in Chinese children. Carriers of the A allele had higher risk for obesity (OR:1.79, P=0.004). The BMI Z-score of children with AA/AT genotype were significantly higher than that TT carriers. |
| Keller K. et al., 2010.^113^ | Cohort | 72 children | 4 to 6 y | Ethnically diverse (African American, Hispanic, White, Asian/East, and others) | *PROP* TAS2R38  A49P | Pyrosequencing | BMI z-score | OR, 95% CI | *PROP* nontaster males had higher BMI z-scores than taster males and females in both groups (P<0.05),  Results suggest that the TAS2R38 variation, PROP phenotype, and sex interact to impact obesity risk in children. |
| Rigoli L. et al., 2010.^114^ | Cohort | 320 children (103 with overweight,  30 with obesity and  187 without obesity). | Overweight  10.5 y  Obesity  9 y  Without obesity  7.8 y | Italy | *CART* A1475G  AA1457 | PCR | BMI | OR, 95% CI | The AA1457 SNP showed no significant association with overweight/obesity. |
| Mong J. et al., 2010.^115^ | Cohort | 981 adolescents | 16±2 y | China | *GHR*  17 SNPs | MALDI-TOF mass spectroscopy on a Sequenom MassARRAY platform | BMI and body composition | OR, 95% CI | There was significant association between rs4410646 and the body composition (OR=0.208, P=0.0044). CC-carriers had lower BMI, %body fat, waist and hip circumferences than AC/AA-carriers. |
| Lee H. et al., 2010.^116^ | Cohort | 711 children | 7 to 10 y | Korean | *FTO*  rs9939973  rs9939609 | Illumina Golden Gate genotyping system | BMI, obesity and overweight | OR, 95% CI | A significant risk association between rs9939973 and rs9939609 on *FTO* with BMI-overweight in children (OR=1.47, p=0.025; OR=1.53, p=0.023, respectively). |
| Riestra P. et al., 2010.^117^ | Cohort | 880 children and adolescents | 12 to 16 y | Spain | *LEP*  G-2548A | Allelic discrimination | Weight, BMI, waist circumference, hip circumference, waist-to-hip ratio, body composition | OR, 95% IC | Male AA-carriers had significantly lower mean hip circumference than GG carriers. In girls, BMI and HC were significantly lower in AA carriers compared with GG. The frequency of the A allele was significantly lower (χ2:4.58) in overweight-obese than normal weight group. |
| Pigeyre M. et al., 2010.^118^ | Cross-sectional study | 1,144 adolescents | 12 to 17 y | Europe | *NMB*  rs1107179 rs17598561 rs3809508  rs1051168 | GoldenGate technology | BMI | OR, 95% CI | The rs3809508-TT was more frequent in obese than healthy adolescents (8.6% vs 3.1%) and the risk of obesity (OR:2.85). Moreover, TT carriers had higher BMI (22.8±4.4 kg m^–2^ vs 21.3±3.7 kg m^– 2^), waist circumference (75.8±9.7cm vs 72.2±9.3 cm), waist-to-hip ratio (0.84±0.14 vs 0.79±0.07) and waist-to-height ratio (0.47±0.06 vs 0.44±0.5) than C-allele carriers. |
| Szopa M. et al., 2010.^119^ | Cohort | 1,155 adolescents from HELENA study | 14.8±1.4 y | Europe | *INSIG1*  rs10258075 rs1128636  rs9692071  rs9690040  rs10271719  rs9770068 | GoldenGate technology | BMI and waist circumference | OR, 95% CI | No association was found between obesity and the analysed SNPs in the HELENA children study. |
| Den Hoed M. et al., 2010.^120^ | Cohort | 1,252 children and 790 adolescents | Children 9.7±0.4 y  Adolescents  15.5±0.5 y | European | Seventeen variants *in NEGR, ETV5*  *SEC16B*  *LYPLAL1*  *TMEM18*  *GNPDA2*  *TFAP2B*  *MSRA*, *BDNF*  *MTCH2*  *BCDIN3D*  *NRXN3*  *SH2B1*  *FTO*, *MC4R*  *KCTD15* | Allelic discrimination | BMI, sum of skinfolds and waist circumference | β-coefficient, 95% CI | Effect sizes for BMI tended to be more pronounced in children and adolescents than reported in adults for variants in or near *SEC16B*, *TMEM18*, and *KCTD15*, (0.028 – 0.035 SD/allele higher) and less pronounced for rs925946 in *BDNF* (0.028 SD/allele lower). Each additional effect allele in the GPS-17 was associated with an increase in BMI, sum of skinfolds, and waist circumference. |
| Bokor S. et al., 2010.^121^ | Case-control | 646 adolescents  (307 with obesity and  339 without obesity)  Validation cohort: 1,151 adolescents | Group 1  13 to 17 y  Validation cohort  12 to 18 y | European | *CD36*  rs3211867  rs3211883  rs3211908  rs1527483  rs1527479  rs3211816  rs3211931 | GoldenGate technology | BMI, body fat % | OR, 95% CI | The four SNPs (rs3211867, rs3211883, rs3211908, and rs1527483) were associated with increased risk of obesity (OR:1.96, *P*=0.003; OR:1.73, *P*=0.007; OR:2.42, *P*=0.0005 and OR:1.95, *P*=0.003, respectively). The same four SNPs were associated with higher BMI and BF% in the validation study. |
| Wang D. et al., 2010.^122^ | Case-control | 2,012 adolescents from ALIR cohort  1,093 adolescents from CPOOA cohort | ALIR cohort  14-17 y  CPOOA cohort  7-18 y | China | *MC4R*  V103I | PCR with restriction fragment length polymorphism assay | BMI | OR, 95% IC | No association was found between the V103I variant and obesity-related phenotypes. However, individuals with I103 allele had a 21% lower risk for obesity (OR=0.79, *P*<0.0001). |
| Vrang N. et al., 2010.^123^ | Cohort | 813 children with obesity | Not specified | French | *BLCAP*  48 SNPs  Including:  rs6090836  rs6019102 | Multiplex PCR target amplification and allelic discrimination | Not specified | OR, 95% CI | The rs6090836 and the rs6019102 SNPs on *BLCAP* were associated with the risk of childhood obesity (OR:1.14, P=0.041 and OR:1.26, P=0.0089, respectively). |
| Liem E. et al., 2010.^124^ | Cohort | 1,275 adolescents | 16.2±0.7 y | Dutch | *INSIG2*  rs7566605  *FTO*  rs9939609  *MC4R*  rs17782313  rs17700633 | Illumina BreadStation 500 platform | BMI  Skinfold | OR, 95% CI and  β-coefficient ±SE | The *FTO*-rs9939609 variant was associated with sex-specific BMI (β:0.11), sum of skinfold thicknesses (β:0.12), percentage body fat (β:0.11), waist circumference (β:0.11), and overweight (OR:1.34) at age 16 y.  Variants near the MC4R gene were associated with BMI (β:0.11). |
| Morandi A. et al., 2010.^125^ | Cohort | 1,852 children and adolescents | 11.68 y | Italy  France | *ADIPOQ*  rs17300539 | Light-Cycler technology | BMI | OR, 95% CI | The rs17300539 GA+AA carriers showed higher BMI (β=0.97, P=0.015) and higher prevalence of obesity (OR=1.35, P=0.015) than GG carriers. |
| Kring S. et al., 2009.^126^ | Cohort | 429 children with severe obesity | 7 to13 y | Denmark | *MC4R*  rs17782313  rs17700633 | Allelic discrimination | BMI | OR, 95% CI | A significant risk association was observed between the *MC4R* variants with BMI during childhood (rs17782313, 13y: OR:1.12, P=0.04 and rs17700633, 10y: OR:1.17, P=0.005 and 13y: OR:1.17, P=0.005, respectively). |
| Moleres A. et al., 2009.^127^ | Cohort | 504 adolescents | 13 to 18 y | Spain | *IL6*  -174G/C | Allelic discrimination | Skinfolds and body fat mas | β-coefficient ± SE | No differences between genotypes were observed in anthropometric values and body composition measurements. |
| Hu P. et al., 2009.^128^ | Cohort | 200 children | 8.2±3.8 y | China | *APOB*  Xbal  EcoRI  MspI | PCR-RFLP | BMI | Mean±SD | *APOB* XbaI and EcoRI may serve as potential genetic markers affecting BMI in children. No significant differences in BMI were found according to *APOB* MspI. |
| Pyrzak B. et al., 2009.^129^ | Case- Control | 142 children (101 with obesity and  41 healthy) | 12 to 18 y | Poland | *LEPR*  Gln223Arg | PCR-RFLP | BMI, waist circumference and skinfolds. | Mean ± SD | In children with obesity, there were not observed association of the *LEPR* Gln223Arg gene polymorphism with obesity. |
| Johansson L. et al., 2009.^130^ | Case-control | 957 adolescents (466 with obesity and 491 without obesity). | 10-18 y | Stockholm | *PNPLA 1*  rs9380559  rs12212459  rs1467912  rs4713951  rs10947600  rs12199580  *PNPLA3*  rs139051  rs12483959  rs2072907 | Allelic discrimination | BMI | OR, 95% CI | The *PNPLA1* rs9380559-SNP was associated with risk of obesity (OR=1.42, P=0.038). On the contrary, rs12212459, rs1467912, rs4713951, rs10947600, and rs12199580 variants that were inversely associated with BMI (OR=0.7, P_adj_<0.05). The variants in *PNPLA3* showed a protective association with obesity (OR=0.7, P=0.023). |
| Lappalainen S. et al., 2009.^131^ | Cohort | 170 children | 7.5±0.9 y | Finnish | *FTO*  rs9939609  *TCF7L2*  rs7903146  rs12255372 | Allelic discrimination | Weight  BMI | Mean ± SD | Risk variant at *FTO* rs9939609 was associated with higher weight-for-height in healthy children (P=0.001). |
| Popko K. et al., 2009.^132^ | Case- control | 105 adolescents (50 with obesity and 55 without obesity) | 10 to 17 y | Poland | *IL-1 β*  C3954T  *IL-6*  G174C  TNF-α  G308A | PCR-RFLP | BMI | OR, 95% CI | The G174C in *IL-6* and G308A in *TNF-α* was associated with risk of obesity. A-G308A allele was more frequent in the group with obesity than in the control (P=0.04). The presence of allele C in promoter region of *IL-6* gene was more frequent in children with obesity and connected with an increase in the sum of 10 skin fold thickness measurements (P=0.03). |
| Dedoussis G. et al., 2009.^133^ | Cohort | 794 adolescents | 10-12 y | Greek | *PPARγ*  rs1801282 | iPLEX MassARRAY platform (Sequenom) | BMI | β-coefficient ± SE | The presence of the Ala allele in boys was a nominally significant predictor of obesity indices, including skinfolds (triceps: β:–2.3, p=0.032; subscapular: β:–2.3, p=0.04) after adjusting for potential covariates. |
| Morandi A. et al., 2009.^134^ | Case-control | 865 children (453 without obesity, 243 with overweight  169 with obesity) | 10.7±1.8 y | Italy | *ENPP1*  K121Q  IVS20delT-11  A/G+1044TGA | Allelic discrimination | BMI | OR, 95% CI | The Q variant of *K121Q* showed a negative association with overweight and obesity under both additive (OR=0.74, P=0.030) and recessive (OR=0.32, P=0.035) modes of inheritance. |
| Cauchi S. et al., 2009.^135^ | Cohort | 4,762 adolescents | 7-16 y | Finnish | *MC4R*  rs17782313  *FTO*  rs1421085 | Allelic discrimination | BMI and fat mass | OR, 95% CI | Each *FTO* and *MC4R* risk allele increased obesity incidences by 24% (P=0.02) and 21% (P=0.02), respectively. The rs17782313-C allele was associated with obesity and fat mass deposition in males than in females (P=0.003 and P=0.03, respectively). |
| Vimaleswaran K. et al., 2009.^136^ | Cohort | 2,003 children and adolescents | Children 9.6±0.4 y  Adolescents  15.5±0.5 y | Denmark  Estonia | *INSIG2*  rs7566605 | Allelic discrimination | BMI | OR, 95% CI | There was no association with overweight, obesity or waist circumference. There was no gender-specific, age-group-specific, or country-specific effects. |
| Eisenmann J. et al., 2009.^137^ | Cohort | 152 children | 3-12 y | United States | *ACE*  rs4341  rs4343 | PCR-RFLP test | BMI | OR, 95% CI | Body mass, BMI, and fat-free mass were significantly higher in the *ACE* D- carriers compared to the II group (p<0.05). |
| Bottcher Y. et al., 2009.^138^ | Cohort | 1,907 children  Replication cohort: 900  Sorbs, 1,029  schoolchildren and 270 children with obesity. | 6-17 y | Germany | *BMPR1A*  21 SNPs included:  rs7095025  rs11202222  rs10788528 rs7922846 | Allelic discrimination | BMI | OR, 95% CI | The SNPs rs7095025, rs11202222, rs10788528, and rs7922846 were associated with risk of obesity (P< 0.05). For the SNPs rs7095025, rs11202222, and rs10788528, the association with risk of obesity was confirmed in the independent cohort of Sorbs (P=0.005). |
| García E. et al., 2008.^139^ | Cohort | 843 children  ALSPAC | 7 y | UK | *GHSR* rs572169 rs495225 rs2232169 | MassEXTEND (hME) assay | Body weight, BMI | Non specified | No association between the analyzed SNPs and anthropometric measures were found. |
| Jacobson J. et al., 2008.^140^ | Case-control | 962 children and adolescents  (450 with severe obesity and 512 without obesity). | Obesity group  12.6 ±3.3 y  Control group  17.1 ±0.8 y | Sweden | *FTO*  rs16952624  rs7499606  rs9939609  rs8051591  *KIAA1005*  rs2111119  rs2302667  rs3213758  rs4784320 | Allelic discrimination | BMI | OR, 95% CI | *FTO* showed significant association with several obesity related traits while SNPs in KIAA1005 did not. When stratified by gender, the *FTO* variant rs9939609 showed association with obesity and BMI among girls (P=0.006 and P=0.004, respectively) but not among boys. |
| Grant S. et al., 2008.^141^ | Case-control | 2,688 Caucasian children (418 with obesity and 2,270 without obesity).  2,2002 adolescents from African-Americans  (578 with obesity and 1,424 without obesity). | 2-18 y | Caucasians and African Americans | *FTO*  rs8050136 rs3751812 | Genome-wide SNP genotyping  using the Illumina Infinium^TM^ II HumanHap550 BeadChip  technology | BMI | OR, 95% CI | The rs8050136 and rs3751812 variants in *FTO* shown a higher risk for obesity (OR=1.26; P=0.0022 and OR=1.26, P=0.0022, respectively) in Caucasians cohort. The rs8050136 was not statistically significant in the cohort of African-Americans. |
| Lagou V. et al., 2008.^142^ | Cohort | 2,102 children | 1-6 y | Greek | *PPARγ*  rs1801282 rs3856806 *PPARδ*  rs2016520 | PCR-RFLP | BMI and skinfolds | Mean, 95% CI | The rs1801282 and rs3856806 polymorphisms were associated with increased adiposity during early childhood in a gender- and  age-specific manner. |
| Wang H. et al., 2008.^143^ | Case-control | 937 adolescents from ALIR cohort  (151 without obesity, 400 overweight and 386 with obesity)  1,103 adolescents from CPOOA cohort  (456 without obesity, 325 overweight and 322 with obesity) | 14.8±0.7 y | China | *INSIG2*  rs7566605 | PCR-RFLP | BMI | OR, 95% CI | *INSIG2* rs7566605 variant was not  associated with Chinese childhood obesity in the two independent cohorts. |
| Ochoa M. et al, 2007.^144^ | Case–control | 363 children and adolescents  (193 with obesity and 170 without obesity) | 6–18 y | Spanish | *UCP2–UCP3* rs659366  rs1800849 | Genotype and allele frequencies were estimated for each polymorphism | BMI | OR, different logistic regression models | The individual analysis of both polymorphisms was not associated with obesity. However, the rs659366-rs1800849) haplotype was significantly associated with obesity and its presence in the control group increased about nine times the insulin resistance risk. |
| Korner A. et al., 2007.^145^ | Cohort | 2,500 children and adolescents from Leipzig cohort.  205 obesity cohort  918 from Pima Indin cohort | 6-17 y | Caucasian | *FAS*  Val1483lle | Taqman SNP Genotyping Assay | BMI and waist-to-hip-ratio | mean±s.e.m | There was no effect of the Val1483lle on BMI in the whole normal population. However, a significant interaction effect between sex and genotype (P=0.004) was found. |
| [Siddiq](https://pubmed.ncbi.nlm.nih.gov/?size=200&term=Siddiq+A&cauthor_id=17235527) A. et al.,2007.^146^ | Case–control | 161 children with severe obesity | 12±0.12 y | Germany | *NPY2R*  rs9770242  *NPY*  rs4730153 | TaqMan allelic discrimination assay | Normal distribution of BMI | Differences in genotype frequencies were compared using logistic regression between children with obesity and without obesity | There were associations for NPY2R and PYY gene variants with obesity and none for PPY variants. A rare variant of the NPY2R gene showed evidence of co-segregation with obesity. A significant association was observed for SNP rs1047214 in NPY2R with an increase in WHR in children with severe obesity |
| Tobias J. et al., 2007.^147^ | Cross-sectional | 3,097 children from ALSPAC cohort | 11 y | Caucasian | ESR1  rs9340799  rs2234693  rs7757956 | Allelic discrimination | Fat mass | OR, 95% IC | The rs7757956 was associated with fat mass (*P=*0.002). Total body fat mass was reduced by 6% in children with TA/AA genotypes, and risk of being overweight was decreased by 20%. This genetic effect appeared to interact with puberty in girls (*P<*0.05 for interaction). |
| Korner A. et al., 2007.^148^ | Cohort | 738 children and adolescent from group 1  205 children and adolescents with obesity from group 2 | Group 1  7.3-16.9 y  Group 2  2.8-17.7 y | Germany | *FAS*  Val1483lle | Allelic discrimination | BMI | Mean ± s.e.m | A significant interaction between sex and genotype was found. Boys carriers of Ile/Val genotype had a lower BMI compared to Val/Val carriers (-0.36 ±0.29 vs 0.09±0.05, P<0.05), while an opposite effect was observed in girls (0.48±0.19 vs 0.09±0.05, P<0.05). |
| Ghoussaini M. et al, 2007.^149^ | Case–control | 2,029 children  (628 with severe obesity and 1,401 without obesity) | 5 y | France | 20 SNPs in *MCHR2* | Direct sequencing | BMI and WHR | OR, 95% IC | Results suggested that *MCHR2* is not a major contributor to polygenic obesity and supported a modest effect of the A76A SNP on food intake abnormalities in childhood. |
| Bottcher Y. et al., 2006.^150^ | Cohort | 712 children from Leipzig cohort  492 children from Lean cohort  205 children from Obesity Leipzing  195 children from Datteln cohort | Leipzig  12±3 y  Lean  2±0.12 y  Obesity Leipzing  11±0.24 y  Datteln  11.2±0.1 y | Germany | *ENPP1*  K121Q  IVS20delT-11  A/G+1044TGA | Allelic discrimination | BMI | OR, 95% CI | The 121Q allele conferred a higher risk of obesity in both additive (OR=1.82, P=0.0005) and recessive mode of inheritance (OR=1.85, P=0.0009) in Leipzing children with obesity compared with children without obesity.  No association of childhood obesity with IVS20delT-11 or A/G+1044TGA was found. |
| Herbert A. et al., 2006.^151^ | Cohort | 368 children | 13.7±3 y | Germany | *INSIG2*  rs7566605 | First: GWAS  Second: Genetic models to predict phenotypes.  Third: Allelic discrimination | BMI | Mean±SD | The rs7566605-C allele is associated with BMI from an early age (GG=31.73 ± 6.29 vs CC =31.95 ±6.05, P=0.0017). |
| Vogels N. et al., 2006.^152^ | Cohort | 105 children  (89 lean subjects and 16 subjects with overweight) | 11 –14 y | Dutch | *PPAR-γ2*  Pro12Ala  GRL -G/C  CNTF -G/A | Allelic discrimination | BMI | Mean ±SD | No significant associations between overweight and the different genotypes of the PPAR-γ, GRL, and CNTF genes were observed in participants. |
| Ochoa M. et al., 2006.^153^ | Case -control | 330 children and adolescents  (165 with obesity and  165 control) | 5-18 y | Spain | *ADRB2*  Gln27Glu | PCR-RFLP | BMI | OR, 95%CI | A higher risk of obesity was found in girls carrying 27Glu carrier allele of the ADRB2 gene (OR=1.95; 95% CI=1.02-3.70), but no association was found among boys. |
| Li S. et al., 2006.^154^ | Cohort | 1,331 children and adolescents | 4 - 17 y | USA | *LPL*  Ser447Stop  *ADRB1*  Arg389Gly | Allelic discrimination | BMI | OR, 95%CI | The combination between the *ADRB1* Arg389Gly and *LPL* Ser447Stop polymorphism was associated with a reduced risk for developing obesity from childhood to adulthood. |
| Marti A. et al., 2006.^155^ | Case -control | Group 1: 370 participants (185 with obesity and 185 control).  Group 2: 124 participants with extreme obesity. | 10-12 y | Group 1: Caucasians (98.4%) and non- Caucasians (1.6%).  Groups 2: Germany | *GRL*  N363S | PCR-RFLP | BMI | OR, 95%CI | The meta-analysis revealed a higher BMI with an overall estimation of 0.18 kg/m^2^ (95% CI:0.004 to 0.35) for homo-/heterozygous carriers of the 363S allele of the *GRL* gene in comparison to non-carriers. |

*ABCA1*: ATP Binding Cassette Subfamily A Member 1; *ABCC8*: ATP Binding Cassette Subfamily C Member 8; *ACE*: Angiotensin I Converting Enzyme; *ACTN3*: Actinin Alpha 3; *ADAMTS9*: ADAM Metallopeptidase With Thrombospondin Type 1 Motif 9; *ADCY3*: Adenylate Cyclase 3; *ADIPOQ*: Adiponectin, C1Q And Collagen Domain Containing; *ADRB1*: Adrenoceptor Beta 1; *ADRB2*: Adrenoceptor Beta 2; *ADRB3*: Adrenoceptor Beta 3; *AGTR1*: Angiotensin II Receptor Type 1; AGТ: Angiotensinogen; *AMD1*: Adenosylmethionine Decarboxylase 1; *ANKK1*: Ankyrin Repeat And Kinase Domain Containing 1; *APOA5*: Apolipoprotein A5; *APOB*: Apolipoprotein B; *BCDIN3D*: BCDIN3 Domain Containing RNA Methyltransferase; *BCHE*: Butyrylcholinesterase; *BDNF*: Brain Derived Neurotrophic Factor; *BLCAP*: BLCAP Apoptosis Inducing Factor; *BMPR1A*: Bone Morphogenetic Protein Receptor Type 1A; *CAT*: Catalase; *CD36:* CD36 Molecule (CD36 Blood Group); *CDH12*: Cadherin 12; *CDKAL1*: CDK5 Regulatory Subunit Associated Protein 1 Like 1; *CDKN2B*: Cyclin Dependent Kinase Inhibitor 2B; *CETP*: Cholesteryl Ester Transfer Protein; *CLOCK*: Clock Circadian Regulator; *CRP*: C-Reactive Protein; *DNM3*: Dynamin 3; DRD2: Dopamine Receptor D2; *ELOVL2*: ELOVL Fatty Acid Elongase 2; *ELP3*: Elongator Acetyltransferase Complex Subunit 3; *ENPP1*: Ectonucleotide Pyrophosphatase/Phosphodiesterase 1; *ESR1*: Estrogen Receptor 1; *ETS2*: ETS Proto-Oncogene 2, Transcription Factor; ETV5: *ETS* Variant Transcription Factor 5; FAAH: Fatty Acid Amide Hydrolase; *FABP2*: Fatty Acid Binding Protein 2; *FADS1*: Fatty Acid Desaturase 1; *FAIM2*: Fas Apoptotic Inhibitory Molecule 2; *FAS*: Fas Cell Surface Death Receptor; *FER1L4*: Fer-1 Like Family Member 4 (Pseudogene) ; *FIGN*: Fidgetin, Microtubule Severing Factor; *FOXO3*: Forkhead Box O3; *FPGT*: Fucose-1-Phosphate Guanylyltransferase; *FTO*: FTO Alpha-Ketoglutarate Dependent Dioxygenase; *GBE1*: 1,4-Alpha-Glucan Branching Enzyme 1; *GHR*: Growth Hormone Receptor; *GHRL*: Ghrelin And Obestatin Prepropeptide; *GHSR*: Growth Hormone Secretagogue Receptor; *GIPR*: Gastric Inhibitory Polypeptide Receptor; *GNPDA2*: Glucosamine-6-Phosphate Deaminase 2; *GPR61*: G Protein-Coupled Receptor 61; *GRB14*: Growth Factor Receptor Bound Protein 14; *GRP*: Gastrin Releasing Peptide; *HHEX*: Hematopoietically Expressed Homeobox; *HNF4A*: Hepatocyte Nuclear Factor 4 Alpha; *HOXB5*: Homeobox B5; *HOXC13*: Homeobox B13; *HSD11B1*: Hydroxysteroid 11-Beta Dehydrogenase 1; *HSD11B2*: Hydroxysteroid 11-Beta Dehydrogenase 2; *IL-1:* Interleukin 1; *IL-6*: Interleukin 6; *IL-6R*: Interleukin 6 Receptor; *INSIG1*: Insulin Induced Gene 1; *INSIG2*: Insulin Induced Gene 2; *IRS1*: Insulin Receptor Substrate 1; *IRX3*: Iroquois Homeobox 3; *ITLN1*: Intelectin 1; *ITPR2*: Inositol 1,4,5-Trisphosphate Receptor Type 2; *KCTD15*: Potassium Channel Tetramerization Domain Containing 15; *KREMEN1*: Kringle Containing Transmembrane Protein 1; *LCT:* Lactase; *LEP*: Leptin; *LEPR*: Leptin receptor; *LIPC*: Lipase C, Hepatic Type; *LMX1B*: LIM Homeobox Transcription Factor 1 Beta; *LPL*: Lipoprotein Lipase; *LRP1B*: LDL Receptor Related Protein 1B; *LYPLAL1*: Lysophospholipase Like 1; *MAF*: MAF BZIP Transcription Factor; *MAOA*: Monoamine Oxidase A; *MAP2K5*: Mitogen-Activated Protein Kinase Kinase 5; *MC3R:* Melanocortin 3 Receptor; *MC4R*: Melanocortin 4 Receptor; *MCHR2*: Melanin Concentrating Hormone Receptor 2; *MFAP3*: Microfibril Associated Protein 3; *MGAT1*: Alpha-1,3-Mannosyl-Glycoprotein 2-Beta-N-Acetylglucosaminyltransferase; MIR148A: MicroRNA 148a; MRPS33P4: Mitochondrial Ribosomal Protein S33 Pseudogene 4; MSRA: Methionine Sulfoxide Reductase A; *MTCH2*: Mitochondrial Carrier 2; *MTHFS*: Methenyltetrahydrofolate Synthetase; *MTNR1B:* Melatonin Receptor 1B; *MTR: 5*-Methyltetrahydrofolate-Homocysteine Methyltransferase; *MTRR*: 5-Methyltetrahydrofolate-Homocysteine Methyltransferase Reductase; *NAMPT*: Nicotinamide Phosphoribosyltransferase; *NEGR1*: Neuronal Growth Regulator 1; *NFE2L3:* NFE2 Like BZIP Transcription Factor 3; *NISCH*: Nischarin; NMB: Neuromedin B; *NPC1*: NPC Intracellular Cholesterol Transporter 1; *NPY*: Neuropeptide Y; *NPY2R:* Neuropeptide Y Receptor Y2; *NRXN3:* Neurexin 3; *NT5C2*: 5'-Nucleotidase, Cytosolic II; *NUCB2*: Nucleobindin 2; *OLFM4*: Olfactomedin 4; *OPRM1*: Opioid Receptor Mu 1; OR: Odd ratio; *PACS1*: Phosphofurin Acidic Cluster Sorting Protein 1; PCK1: Phosphoenolpyruvate Carboxykinase 1; PCSK1: Proprotein Convertase Subtilisin/Kexin Type 1; PNPLA1: Patatin Like Phospholipase Domain Containing 1; *PNPLA3*: Patatin Like Phospholipase Domain Containing 3; *PON1*: Paraoxonase 1; *PPARγ:* Peroxisome Proliferator Activated Receptor Gamma; *PRKCH*: Protein Kinase C Eta; *PRKD1*: Protein Kinase D1; *PROP1*: PROP Paired-Like Homeobox 1; *QPCTL*: Glutaminyl-Peptide Cyclotransferase Like; *RAB27B*: RAB27B, Member RAS Oncogene Family; *RABEP1:* Rabaptin, RAB GTPase Binding Effector Protein 1*; RASA2:* RAS P21 Protein Activator 2; RBP4: Retinol Binding Protein 4; *RMST*: Rhabdomyosarcoma 2 Associated Transcript; *ROPN1L*: Rhophilin Associated Tail Protein 1 Like; *RPTOR*: Regulatory Associated Protein Of MTOR Complex 1; RSPO3: R-Spondin 3; SEC16B: SEC16 Homolog B, Endoplasmic Reticulum Export Factor; SERPINA12: Serpin Family A Member 12*; SERPINF1:* Serpin Family F Member 1; *SH2B1:* SH2B Adaptor Protein 1; *SHBG*: Sex Hormone Binding Globulin; *SIRT1:* Sirtuin 1; SLC30A8: Solute Carrier Family 30 Member 8; SLC6A4: Solute Carrier Family 6 Member 4; SOCS3: Suppressor Of Cytokine Signaling 3; STAB1: Stabilin 1; *STK33:* Serine/Threonine Kinase 33; *TBX15:* T-Box Transcription Factor 15; *TCF7L2*: Transcription Factor 7 Like 2; *TCN2*: Transcobalamin 2; *TFAP2B*: Transcription Factor AP-2 Beta; *TMEM18:* Transmembrane Protein 18; *TMEM212:* Transmembrane Protein 212; *TNF-α:* Tumor Necrosis Factor Alpha*; TNNI3K*: TNNI3 Interacting Kinase; *TRIM66:* Tripartite Motif Containing 66*; TUB:* TUB Bipartite Transcription Factor; *UCP1:* Uncoupling Protein 1; *UCP2:* Uncoupling Protein 2; *UCP3:* Uncoupling Protein 3; U*SP37*: Ubiquitin Specific Peptidase 37; *VEGFA:* Vascular Endothelial Growth Factor A; *ZNF608:* Zinc Finger Protein 608 and *ZNRF3:* Zinc And Ring Finger 3.

**Reference**

1. Adiyeva M., N. Aukenov, A. Nurzhanova, *et al.* 2023. The effect of AGTR1, AGТ, LPL, ADRB2 gene polymorphisms on central obesity in adolescents of the Kazakh population. *Bratisl Lek Listy* **124**: 53–58. https://doi.org/10.4149/BLL_2023_008

2. Manco L., A.M. Machado-Rodrigues & C. Padez. 2022. Association study of common functional genetic polymorphisms in SLC6A4 (5-HTT) and MAOA genes with obesity in portuguese children. *Arch Physiol Biochem* **128**: 1510–1515. https://doi.org/10.1080/13813455.2020.1779312

3. Reuter É.M., C.P. Reuter, J.F. de Castro Silveira, *et al.* 2021. FTO gene polymorphism and longitudinal changes in nutritional/obesity status in children and adolescents: Schoolchildren’s health cohort study. *Eur J Pediatr* **180**: 3325–3333. https://doi.org/10.1007/S00431-021-04120-0

4. Walia G.K., S. Saini, P. Vimal, *et al.* 2021. Association of MC4R (rs17782313) gene polymorphism with obesity measures in Western India. *Diabetes Metab Syndr* **15**: 661–665. https://doi.org/10.1016/J.DSX.2021.03.013

5. Molina-Luque R., N. Ulloa, M. Romero-Saldaña, *et al.* 2021. Association between the FTO SNP rs9939609 and Metabolic Syndrome in Chilean Children. *Nutrients* **13**:. https://doi.org/10.3390/NU13062014

6. Kroll C., D.R. Farias, G. Kac, *et al.* 2021. Adiponectin and leptin gene variants and their effects on body weight trajectories in children from birth to 6 years of age: the PREDI Study. *Br J Nutr* **125**: 241–250. https://doi.org/10.1017/S0007114520002780

7. Enciso-Ramírez M., Z. Reyes-Castillo, M.A. Llamas-Covarrubias, *et al.* 2021. CD36 gene polymorphism -31118 G > A (rs1761667) is associated with overweight and obesity but not with fat preferences in Mexican children. *Int J Vitam Nutr Res* **91**: 513–521. https://doi.org/10.1024/0300-9831/A000656

8. Raskiliene A., A. Smalinskiene, V. Kriaucioniene, *et al.* 2021. Associations of MC4R, LEP, and LEPR Polymorphisms with Obesity-Related Parameters in Childhood and Adulthood. *Genes (Basel)* **12**:. https://doi.org/10.3390/GENES12060949

9. Quevedo Alves F., C.P. Reuter, I. Neumann, *et al.* 2022. Relationship between rs9939609 FTO polymorphism with waist circumference and body fat is moderated by ponderal index at birth in youth. *Am J Hum Biol* **34**:. https://doi.org/10.1002/AJHB.23575

10. Kulaeva E.D., V. V. Volchik, O. V. Bocharova, *et al.* 2021. Association of SNPs in Lipid Metabolism Gene Single Nucleotide Polymorphism with the Risk of Obesity in Children. *Genet Test Mol Biomarkers* **25**: 419–425. https://doi.org/10.1089/GTMB.2020.0343

11. Maguolo A., C. Zusi, A. Giontella, *et al.* 2021. Influence of genetic variants in FADS2 and ELOVL2 genes on BMI and PUFAs homeostasis in children and adolescents with obesity. *Int J Obes (Lond)* **45**: 56–65. https://doi.org/10.1038/S41366-020-00662-9

12. García-Rodríguez M.H., B.I. Peña-Espinoza, M. De Los Angeles Granados-Silvestre, *et al.* 2020. Association of the T130I Variant of the HNF4A Gene with Metabolic Syndrome and Its Components in Mexican Children. *Metab Syndr Relat Disord* **18**: 479–484. https://doi.org/10.1089/MET.2020.0024

13. López-Rodríguez G., A. Estrada-Neria, T. Suárez-Diéguez, *et al.* 2020. Common polymorphisms in MC4R and FTO genes are associated with BMI and metabolic indicators in Mexican children: Differences by sex and genetic ancestry. *Gene* **754**:. https://doi.org/10.1016/J.GENE.2020.144840

14. Costa-Urrutia P., C. Abud, V. Franco-Trecu, *et al.* 2020. Effect of 15 BMI-Associated Polymorphisms, Reported for Europeans, across Ethnicities and Degrees of Amerindian Ancestry in Mexican Children. *Int J Mol Sci* **21**:. https://doi.org/10.3390/IJMS21020374

15. Meng Y., B. Lohse & L. Cunningham-Sabo. 2020. Sex modifies the association between the CLOCK variant rs1801260 and BMI in school-age children. *PLoS One* **15**:. https://doi.org/10.1371/JOURNAL.PONE.0236991

16. Ulloa N., M. Villagrán, B. Riffo, *et al.* 2020. [Association between FTO gene rs9939609 and adiposity markers in Chilean children]. *Rev Chil Pediatr* **91**: 371–378. https://doi.org/10.32641/RCHPED.V91I3.1395

17. Carrillo-Venzor M.A., N.R. Erives-Anchondo, J.G. Moreno-González, *et al.* 2020. Pro12Ala PPAR-γ2 and +294T/C PPAR-δ Polymorphisms and Association with Metabolic Traits in Teenagers from Northern Mexico. *Genes (Basel)* **11**: 1–18. https://doi.org/10.3390/GENES11070776

18. Justice A.E., G. Chittoor, E. Blanco, *et al.* 2019. Genetic determinants of BMI from early childhood to adolescence: the Santiago Longitudinal Study. *Pediatr Obes* **14**:. https://doi.org/10.1111/IJPO.12479

19. Manco L., S. Pinho, D. Albuquerque, *et al.* 2019. Physical activity and the association between the FTO rs9939609 polymorphism and obesity in Portuguese children aged 3 to 11 years. *Am J Hum Biol* **31**:. https://doi.org/10.1002/AJHB.23312

20. Pascual-Gamarra J.M., D. Salazar-Tortosa, B. Martinez-Tellez, *et al.* 2019. Association between UCP1, UCP2, and UCP3 gene polymorphisms with markers of adiposity in European adolescents: The HELENA study. *Pediatr Obes* **14**:. https://doi.org/10.1111/IJPO.12504

21. Gajewska J., A. Kuryłowicz, E. Mierzejewska, *et al.* 2020. Are Omentin Rs2274907 and Vaspin Rs2236242 Gene Polymorphisms Related to Body Composition, Lipid Profile and Other Adipokines in Prepubertal Healthy Children? *Endocr Res* **45**: 24–31. https://doi.org/10.1080/07435800.2019.1630842

22. Lee S. 2019. The genetic and epigenetic association of LDL Receptor Related Protein 1B (LRP1B) gene with childhood obesity. *Sci Rep* **9**:. https://doi.org/10.1038/S41598-019-38538-2

23. Cardel M.I., D.J. Lemas, A.M. Lee, *et al.* 2019. Taq1a polymorphism (rs1800497) is associated with obesity-related outcomes and dietary intake in a multi-ethnic sample of children. *Pediatr Obes* **14**:. https://doi.org/10.1111/IJPO.12470

24. Ren D., J.H. Xu, Y. Bi, *et al.* 2019. Association study between LEPR, MC4R polymorphisms and overweight/obesity in Chinese Han adolescents. *Gene* **692**: 54–59. https://doi.org/10.1016/J.GENE.2018.12.073

25. Ferreira Todendi P., A.R. de Moura Valim, E. Klinger, *et al.* 2019. The role of the genetic variants IRX3 rs3751723 and FTO rs9939609 in the obesity phenotypes of children and adolescents. *Obes Res Clin Pract* **13**: 137–142. https://doi.org/10.1016/J.ORCP.2019.01.005

26. Yang Y., X.H. Gao, X.J. Tao, *et al.* 2019. Combined effect of FTO and MC4R gene polymorphisms on obesity in children and adolescents in Northwest China: a case-control study. *Asia Pac J Clin Nutr* **28**: 177–182. https://doi.org/10.6133/APJCN.201903_28(1).0023

27. Liu H.Y., A. Alyass, A. Abadi, *et al.* 2019. Fine-mapping of 98 obesity loci in Mexican children. *Int J Obes (Lond)* **43**: 23–32. https://doi.org/10.1038/S41366-018-0056-7

28. Turcotte M., A. Abadi, J. Peralta-Romero, *et al.* 2019. Genetic contribution to waist-to-hip ratio in Mexican children and adolescents based on 12 loci validated in European adults. *Int J Obes (Lond)* **43**: 13–22. https://doi.org/10.1038/S41366-018-0055-8

29. Muller Y.L., R.L. Hanson, P. Piaggi, *et al.* 2019. Assessing the Role of 98 Established Loci for BMI in American Indians. *Obesity (Silver Spring)* **27**: 845–854. https://doi.org/10.1002/OBY.22433

30. Mărginean C.O., C. Mărginean, M. Iancu, *et al.* 2019. The impact of TNF-α 308G>A gene polymorphism on children’s overweight risk and an assessment of biochemical variables: A cross-sectional single-center experience. *Pediatr Neonatol* **60**: 19–27. https://doi.org/10.1016/J.PEDNEO.2018.03.003

31. Zaharan N.L., N.H. Muhamad, M.Y. Jalaludin, *et al.* 2018. Non-Synonymous Single-Nucleotide Polymorphisms and Physical Activity Interactions on Adiposity Parameters in Malaysian Adolescents. *Front Endocrinol (Lausanne)* **9**:. https://doi.org/10.3389/FENDO.2018.00209

32. Dos Santos Rocha A., R. de Cássia Ribeiro-Silva, G. Nunes de Oliveira Costa, *et al.* 2018. Food Consumption as a Modifier of the Association between LEPR Gene Variants and Excess Body Weight in Children and Adolescents: A Study of the SCAALA Cohort. *Nutrients* **10**:. https://doi.org/10.3390/NU10081117

33. Çöl N., M. Nacak & M. Araz. 2018. Association of melatonin receptor 1 B gene (rs10830963 and rs9192552) polymorphısm with adolescent obesity and related comorbidities in Turkey. *J Int Med Res* **46**: 3086–3096. https://doi.org/10.1177/0300060518772224

34. Almeida S.M., J.M. Furtado, P. Mascarenhas, *et al.* 2018. Association between LEPR, FTO, MC4R, and PPARG-2 polymorphisms with obesity traits and metabolic phenotypes in school-aged children. *Endocrine* **60**: 466–478. https://doi.org/10.1007/S12020-018-1587-3

35. Zandoná M.R., C.N. Sangalli, P.D.B. Campagnolo, *et al.* 2017. Validation of obesity susceptibility loci identified by genome-wide association studies in early childhood in South Brazilian children. *Pediatr Obes* **12**: 85–92. https://doi.org/10.1111/IJPO.12113

36. Dorajoo R., R.T.H. Ong, X. Sim, *et al.* 2017. The contribution of recently identified adult BMI risk loci to paediatric obesity in a Singaporean Chinese childhood dataset. *Pediatr Obes* **12**: e46–e50. https://doi.org/10.1111/IJPO.12175

37. Boyraz M., E. Yeşilkaya, F. Ezgü, *et al.* 2016. Effect of Cytokine Signaling 3 Gene Polymorphisms in Childhood Obesity. *J Clin Res Pediatr Endocrinol* **8**: 452–460. https://doi.org/10.4274/JCRPE.3167

38. Codoñer-Franch P., J. Carrasco-Luna, P. Allepuz, *et al.* 2016. Association of RBP4 genetic variants with childhood obesity and cardiovascular risk factors. *Pediatr Diabetes* **17**: 576–583. https://doi.org/10.1111/PEDI.12339

39. Reuter C.P., M.S. Burgos, J.C. Bernhard, *et al.* 2016. Association between overweight and obesity in schoolchildren with rs9939609 polymorphism (FTO) and family history for obesity. *J Pediatr (Rio J)* **92**: 493–498. https://doi.org/10.1016/J.JPED.2015.11.005

40. Aris I.M., M.T. Tint, A.L. Teh, *et al.* 2016. MC3R gene polymorphisms are associated with early childhood adiposity gain and infant appetite in an Asian population. *Pediatr Obes* **11**: 450–458. https://doi.org/10.1111/IJPO.12086

41. Kaulfers A.M., R. Deka, L. Dolan, *et al.* 2015. Association of INSIG2 polymorphism with overweight and LDL in children. *PLoS One* **10**:. https://doi.org/10.1371/JOURNAL.PONE.0116340

42. Shahid A., S. Rana, S. Mahmood, *et al.* 2015. Role of leptin G-2548A polymorphism in age- and gender-specific development of obesity. *J Biosci* **40**: 521–530. https://doi.org/10.1007/S12038-015-9536-2

43. Kilic U., O. Gok, B. Elibol-Can, *et al.* 2015. SIRT1 gene variants are related to risk of childhood obesity. *Eur J Pediatr* **174**: 473–479. https://doi.org/10.1007/S00431-014-2424-1

44. Pillay V., N.J. Crowther, M. Ramsay, *et al.* 2015. Exploring genetic markers of adult obesity risk in black adolescent South Africans-the Birth to Twenty Cohort. *Nutr Diabetes* **5**:. https://doi.org/10.1038/NUTD.2015.7

45. White M.J., F. Eren, D. Agirbasli, *et al.* 2015. SHBG gene polymorphism (rs1799941) associates with metabolic syndrome in children and adolescents. *PLoS One* **10**:. https://doi.org/10.1371/JOURNAL.PONE.0116915

46. Rask-Andersen M., M. Sällman Almén, J.A. Jacobsson, *et al.* 2015. Determination of obesity associated gene variants related to TMEM18 through ultra-deep targeted re-sequencing in a case-control cohort for pediatric obesity. *Genet Res (Camb)* **97**: e16. https://doi.org/10.1017/S0016672315000117

47. Todendi P.F., E.I. Klinger, M.B. Ferreira, *et al.* 2015. Association of IL-6 and CRP gene polymorphisms with obesity and metabolic disorders in children and adolescents. *An Acad Bras Cienc* **87**: 915–924. https://doi.org/10.1590/0001-3765201520140364

48. Wang H., D. Zhang, J. Ling, *et al.* 2015. Gender specific effect of LIPC C-514T polymorphism on obesity and relationship with plasma lipid levels in Chinese children. *J Cell Mol Med* **19**: 2296–2306. https://doi.org/10.1111/JCMM.12663

49. Fawzy M.S., O. Alhadramy, M.H. Hussein, *et al.* 2015. Functional and Structural Impact of ATP-Binding Cassette Transporter A1 R219K and I883M Gene Polymorphisms in Obese Children and Adolescents. *Mol Diagn Ther* **19**: 221–234. https://doi.org/10.1007/S40291-015-0150-7

50. Hollensted M., T.S. Ahluwalia, C.T. Have, *et al.* 2015. Common variants in LEPR, IL6, AMD1, and NAMPT do not associate with risk of juvenile and childhood obesity in Danes: a case-control study. *BMC Med Genet* **16**:. https://doi.org/10.1186/S12881-015-0253-3

51. Hardman C.A., P.J. Rogers, N.J. Timpson, *et al.* 2014. Lack of association between DRD2 and OPRM1 genotypes and adiposity. *Int J Obes (Lond)* **38**: 730–736. https://doi.org/10.1038/IJO.2013.144

52. Zhu W.F., C.L. Wang, L. Liang, *et al.* 2014. Triglyceride-raising APOA5 genetic variants are associated with obesity and non-HDL-C in Chinese children and adolescents. *Lipids Health Dis* **13**:. https://doi.org/10.1186/1476-511X-13-93

53. Albuquerque D., C. Nóbrega, R. Rodríguez-López, *et al.* 2014. Association study of common polymorphisms in MSRA, TFAP2B, MC4R, NRXN3, PPARGC1A, TMEM18, SEC16B, HOXB5 and OLFM4 genes with obesity-related traits among Portuguese children. *J Hum Genet* **59**: 307–313. https://doi.org/10.1038/JHG.2014.23

54. Marcovecchio M.L., R. Capanna, E. D’Adamo, *et al.* 2014. Association between rs12970134 Near MC4R and adiposity indexes in a homogenous population of Caucasian schoolchildren. *Horm Res Paediatr* **82**: 187–193. https://doi.org/10.1159/000365103

55. Oana M.C., B. Claudia, D. Carmen, *et al.* 2014. The role of IL-6 572 C/G, 190 C/T, and 174 G/C gene polymorphisms in children’s obesity. *Eur J Pediatr* **173**: 1285–1296. https://doi.org/10.1007/S00431-014-2315-5

56. Meng X.R., J.Y. Song, J. Ma, *et al.* 2014. Association study of childhood obesity with eight genetic variants recently identified by genome-wide association studies. *Pediatr Res* **76**: 310–315. https://doi.org/10.1038/PR.2014.88

57. Muller Y.L., M.S. Thearle, P. Piaggi, *et al.* 2014. Common genetic variation in and near the melanocortin 4 receptor gene (MC4R) is associated with body mass index in American Indian adults and children. *Hum Genet* **133**: 1431–1441. https://doi.org/10.1007/S00439-014-1477-6

58. Ng Z.Y., M.K. Veerapen, W.M. Hon, *et al.* 2014. Association of leptin/receptor and TNF-α gene variants with adolescent obesity in Malaysia. *Pediatr Int* **56**: 689–697. https://doi.org/10.1111/PED.12336

59. Ruan L.L., J. Xu, C.L. Wang, *et al.* 2014. Variants of 11β-hydroxysteroid dehydrogenase (HSD11B) gene type 1 and 2 in Chinese obese adolescents. *J Endocrinol Invest* **37**: 565–573. https://doi.org/10.1007/S40618-014-0075-8

60. Shinozaki K., M. Okuda, Y. Hinoda, *et al.* 2014. Fat-mass and obesity-associated gene variant and changes of body mass index from ages 3 to 13 years. *Obes Res Clin Pract* **8**:. https://doi.org/10.1016/J.ORCP.2013.07.005

61. Wu J., J. Xu, Z. Zhang, *et al.* 2014. Association of FTO polymorphisms with obesity and metabolic parameters in Han Chinese adolescents. *PLoS One* **9**:. https://doi.org/10.1371/JOURNAL.PONE.0098984

62. Xu Y., J. Ling, M. Yang, *et al.* 2014. Rs7206790 and rs11644943 in FTO gene are associated with risk of obesity in Chinese school-age population. *PLoS One* **9**:. https://doi.org/10.1371/JOURNAL.PONE.0108050

63. Yang M., Y. Xu, L. Liang, *et al.* 2014. The effects of genetic variation in FTO rs9939609 on obesity and dietary preferences in Chinese Han children and adolescents. *PLoS One* **9**:. https://doi.org/10.1371/JOURNAL.PONE.0104574

64. Zhang M., X. Zhao, H. Cheng, *et al.* 2014. Age- and sex-dependent association between FTO rs9939609 and obesity-related traits in Chinese children and adolescents. *PLoS One* **9**:. https://doi.org/10.1371/JOURNAL.PONE.0097545

65. Ho-Urriola J., I.P. Guzmán-Guzmán, S. V. Smalley, *et al.* 2014. Melanocortin-4 receptor polymorphism rs17782313: association with obesity and eating in the absence of hunger in Chilean children. *Nutrition* **30**: 145–149. https://doi.org/10.1016/J.NUT.2013.05.030

66. Albuquerque D., C. Nóbrega & L. Manco. 2013. Association of FTO polymorphisms with obesity and obesity-related outcomes in Portuguese children. *PLoS One* **8**:. https://doi.org/10.1371/JOURNAL.PONE.0054370

67. Chaves T.J., N. Leite, G.E. Milano, *et al.* 2013. -116A and K BCHE gene variants associated with obesity and hypertriglyceridemia in adolescents from Southern Brazil. *Chem Biol Interact* **203**: 341–343. https://doi.org/10.1016/J.CBI.2012.09.006

68. Chen Y.Y., R.M.E. Chan, K.M.L. Tan, *et al.* 2013. The association of a nucleobindin 2 gene (NUCB2) variant with childhood adiposity. *Gene* **516**: 48–52. https://doi.org/10.1016/J.GENE.2012.12.017

69. Rupérez A.I., O. López-Guarnido, F. Gil, *et al.* 2013. Paraoxonase 1 activities and genetic variation in childhood obesity. *Br J Nutr* **110**: 1639–1647. https://doi.org/10.1017/S0007114513001967

70. Wang L., Q. Yu, Y. Xiong, *et al.* 2013. Variant rs1421085 in the FTO gene contribute childhood obesity in Chinese children aged 3-6 years. *Obes Res Clin Pract* **7**:. https://doi.org/10.1016/J.ORCP.2011.12.007

71. Rupérez A.I., J. Olza, M. Gil-Campos, *et al.* 2013. Are catalase -844A/G polymorphism and activity associated with childhood obesity? *Antioxid Redox Signal* **19**: 1970–1975. https://doi.org/10.1089/ARS.2013.5386

72. Huen K., K. Harley, K. Beckman, *et al.* 2013. Associations of PON1 and genetic ancestry with obesity in early childhood. *PLoS One* **8**:. https://doi.org/10.1371/JOURNAL.PONE.0062565

73. León-Mimila P., H. Villamil-Ramírez, M. Villalobos-Comparán, *et al.* 2013. Contribution of common genetic variants to obesity and obesity-related traits in mexican children and adults. *PLoS One* **8**:. https://doi.org/10.1371/JOURNAL.PONE.0070640

74. Olza J., M. Gil-Campos, R. Leis, *et al.* 2013. Influence of variants in the NPY gene on obesity and metabolic syndrome features in Spanish children. *Peptides (N.Y.)* **45**: 22–27. https://doi.org/10.1016/J.PEPTIDES.2013.04.007

75. Goumidi L., A. Grechez, J. Dumont, *et al.* 2013. Impact of REV-ERB alpha gene polymorphisms on obesity phenotypes in adult and adolescent samples. *Int J Obes (Lond)* **37**: 666–672. https://doi.org/10.1038/IJO.2012.117

76. Wheeler E., N. Huang, E.G. Bochukova, *et al.* 2013. Genome-wide SNP and CNV analysis identifies common and low-frequency variants associated with severe early-onset obesity. *Nat Genet* **45**: 513–517. https://doi.org/10.1038/NG.2607

77. De La Cruz-Mosso U., J.F. Muñoz-Valle, A.B. Salgado-Bernabé, *et al.* 2013. Body adiposity but not insulin resistance is associated with -675 4G/5G polymorphism in the PAI-1 gene in a sample of Mexican children. *J Pediatr (Rio J)* **89**: 492–498. https://doi.org/10.1016/J.JPED.2013.01.004

78. Ntalla I., K. Panoutsopoulou, P. Vlachou, *et al.* 2013. Replication of established common genetic variants for adult BMI and childhood obesity in Greek adolescents: the TEENAGE study. *Ann Hum Genet* **77**: 268–274. https://doi.org/10.1111/AHG.12012

79. Rask-Andersen M., G. Moschonis, G.P. Chrousos, *et al.* 2013. The STK33-linked SNP rs4929949 is associated with obesity and BMI in two independent cohorts of Swedish and Greek children. *PLoS One* **8**:. https://doi.org/10.1371/JOURNAL.PONE.0071353

80. Mejía-Benítez A., M. Klünder-Klünder, L. Yengo, *et al.* 2013. Analysis of the contribution of FTO, NPC1, ENPP1, NEGR1, GNPDA2 and MC4R genes to obesity in Mexican children. *BMC Med Genet* **14**:. https://doi.org/10.1186/1471-2350-14-21

81. Xi B., H. Cheng, Y. Shen, *et al.* 2013. Study of 11 BMI-associated loci identified in GWAS for associations with central obesity in the Chinese children. *PLoS One* **8**:. https://doi.org/10.1371/JOURNAL.PONE.0056472

82. Xi B., Y. Shen, K.H. Reilly, *et al.* 2013. Sex-dependent associations of genetic variants identified by GWAS with indices of adiposity and obesity risk in a Chinese children population. *Clin Endocrinol (Oxf)* **79**: 523–528. https://doi.org/10.1111/CEN.12091

83. Olza J., A.I. Ruperez, M. Gil-Campos, *et al.* 2013. Influence of FTO variants on obesity, inflammation and cardiovascular disease risk biomarkers in Spanish children: a case-control multicentre study. *BMC Med Genet* **14**:. https://doi.org/10.1186/1471-2350-14-123

84. Sällman Almén M., M. Rask-Andersen, J.A. Jacobsson, *et al.* 2013. Determination of the obesity-associated gene variants within the entire FTO gene by ultra-deep targeted sequencing in obese and lean children. *Int J Obes (Lond)* **37**: 424–431. https://doi.org/10.1038/IJO.2012.57

85. Csernus K., G. Pauler, E. Erhardt, *et al.* 2013. Uncoupling protein-2 gene polymorphisms are associated with obesity in Hungarian children. *Acta Paediatr* **102**:. https://doi.org/10.1111/APA.12181

86. Albuquerque D., C. Nõbrega & L. Manco. 2013. The lactase persistence -13910C>T polymorphism shows indication of association with abdominal obesity among Portuguese children. *Acta Paediatr* **102**:. https://doi.org/10.1111/APA.12134

87. Dwivedi O.P., R. Tabassum, G. Chauhan, *et al.* 2012. Common variants of FTO are associated with childhood obesity in a cross-sectional study of 3,126 urban Indian children. *PLoS One* **7**:. https://doi.org/10.1371/JOURNAL.PONE.0047772

88. Chen Y.Y., R.M.E. Chan, K.M.L. Tan, *et al.* 2013. The association of a nucleobindin 2 gene (NUCB2) variant with childhood adiposity. *Gene* **516**: 48–52. https://doi.org/10.1016/J.GENE.2012.12.017

89. De la Cruz-Mosso U., J.F. Muñoz-Valle, L. Salgado-Goytia, *et al.* 2012. Relationship of metabolic syndrome and its components with -844 G/A and HindIII C/G PAI-1 gene polymorphisms in Mexican children. *BMC Pediatr* **12**:. https://doi.org/10.1186/1471-2431-12-41

90. Jacobsson J.A., M. Rask-Andersen, U. Risérus, *et al.* 2012. Genetic variants near the MGAT1 gene are associated with body weight, BMI and fatty acid metabolism among adults and children. *Int J Obes (Lond)* **36**: 119–129. https://doi.org/10.1038/IJO.2011.11

91. Hsieh C.H., Y.J. Hung, L.I. Wu, *et al.* 2012. Interleukin-6 receptor gene 48892 A/C polymorphism is associated with metabolic syndrome in female Taiwanese adolescents. *Genet Test Mol Biomarkers* **16**: 1376–1381. https://doi.org/10.1089/GTMB.2012.0188

92. Wang J., H. Mei, W. Chen, *et al.* 2012. Study of eight GWAS-identified common variants for association with obesity-related indices in Chinese children at puberty. *Int J Obes (Lond)* **36**: 542–547. https://doi.org/10.1038/IJO.2011.218

93. Duran-Gonzalez J., I. Ortiz, E. Gonzales, *et al.* 2011. Association study of candidate gene polymorphisms and obesity in a young Mexican-American population from South Texas. *Arch Med Res* **42**: 523–531. https://doi.org/10.1016/J.ARCMED.2011.10.010

94. Gao L., Z. Zheng, L. Cao, *et al.* 2011. The growth hormone receptor (GHR) exon 3 polymorphism and its correlation with metabolic profiles in obese Chinese children. *Pediatr Diabetes* **12**: 429–434. https://doi.org/10.1111/J.1399-5448.2010.00747.X

95. Horvatovich K., S. Bokor, Á. Baráth, *et al.* 2011. Haplotype analysis of the apolipoprotein A5 gene in obese pediatric patients. *Int J Pediatr Obes* **6**:. https://doi.org/10.3109/17477166.2010.490268

96. Bokor S., A. Meirhaeghe, J.R. Ruiz, *et al.* 2011. Common polymorphisms in six genes of the methyl group metabolism pathway and obesity in European adolescents. *Int J Pediatr Obes* **6**:. https://doi.org/10.3109/17477166.2010.500386

97. Lin F.H., N.F. Chu, C.H. Lee, *et al.* 2011. Combined effect of C-reactive protein gene SNP +2147 A/G and interleukin-6 receptor gene SNP rs2229238 C/T on anthropometric characteristics among school children in Taiwan. *Int J Obes (Lond)* **35**: 587–594. https://doi.org/10.1038/IJO.2010.195

98. Zhu J.F., L. Liang, C.C. Zou, *et al.* 2010. Plasma ghrelin levels and polymorphisms of ghrelin gene in Chinese obese children and adolescents. *Ir J Med Sci* **179**: 345–349. https://doi.org/10.1007/S11845-010-0494-2

99. Bollepalli S., L.M. Dolan, R. Deka, *et al.* 2010. Association of FTO gene variants with adiposity in African-American adolescents. *Obesity (Silver Spring)* **18**: 1959–1963. https://doi.org/10.1038/OBY.2010.82

100. Solaas K., V. Legry, K. Retterstol, *et al.* 2010. Suggestive evidence of associations between liver X receptor β polymorphisms with type 2 diabetes mellitus and obesity in three cohort studies: HUNT2 (Norway), MONICA (France) and HELENA (Europe). *BMC Med Genet* **11**:. https://doi.org/10.1186/1471-2350-11-144

101. Sauber J., J. Grothe, M. Behm, *et al.* 2010. Association of variants in gastric inhibitory polypeptide receptor gene with impaired glucose homeostasis in obese children and adolescents from Berlin. *Eur J Endocrinol* **163**: 259–264. https://doi.org/10.1530/EJE-10-0444

102. Müller T.D., G. Brönner, M. Wandolski, *et al.* 2010. Mutation screen and association studies for the fatty acid amide hydrolase (FAAH) gene and early onset and adult obesity. *BMC Med Genet* **11**:. https://doi.org/10.1186/1471-2350-11-2

103. Wu L., B. Xi, M. Zhang, *et al.* 2010. Associations of six single nucleotide polymorphisms in obesity-related genes with BMI and risk of obesity in Chinese children. *Diabetes* **59**: 3085–3089. https://doi.org/10.2337/DB10-0273

104. Ma L., R.L. Hanson, M.T. Traurig, *et al.* 2010. Evaluation of A2BP1 as an obesity gene. *Diabetes* **59**: 2837–2845. https://doi.org/10.2337/DB09-1604

105. Riestra P., A. García-Anguita, S. Schoppen, *et al.* 2010. Sex-specific association between leptin receptor polymorphisms and leptin levels and BMI in healthy adolescents. *Acta Paediatr* **99**: 1527–1530. https://doi.org/10.1111/J.1651-2227.2010.01877.X

106. Xi B., Y. Shen, M. Zhang, *et al.* 2010. The common rs9939609 variant of the fat mass and obesity-associated gene is associated with obesity risk in children and adolescents of Beijing, China. *BMC Med Genet* **11**:. https://doi.org/10.1186/1471-2350-11-107

107. Vimaleswaran K.S., P.W. Franks, S. Brage, *et al.* 2010. Lack of association between PCK1 polymorphisms and obesity, physical activity, and fitness in European Youth Heart Study (EYHS). *Obesity (Silver Spring)* **18**: 1975–1980. https://doi.org/10.1038/OBY.2010.13

108. Pyrzak B., A. Wisniewska, K. Popko, *et al.* 2010. Association between anthropometric measures of obesity, metabolic disturbances and polymorphism G-308A of the tumor necrosis factor-alpha gene in children. *Eur J Med Res* **15 Suppl 2**: 141–146. https://doi.org/10.1186/2047-783X-15-S2-141

109. Mo J., J. Wu, Z. Sun, *et al.* 2010. Association of PTP1B gene polymorphism with obesity in Chinese children. *Zhong Nan Da Xue Xue Bao Yi Xue Ban* **35**: 915–920. https://doi.org/10.3969/J.ISSN.1672-7347.2010.09.003

110. Ruiz J.R., I. Labayen, F.B. Ortega, *et al.* 2010. Attenuation of the effect of the FTO rs9939609 polymorphism on total and central body fat by physical activity in adolescents: the HELENA study. *Arch Pediatr Adolesc Med* **164**: 328–333. https://doi.org/10.1001/ARCHPEDIATRICS.2010.29

111. Almén M.S., J.A. Jacobsson, J.H.A. Shaik, *et al.* 2010. The obesity gene, TMEM18, is of ancient origin, found in majority of neuronal cells in all major brain regions and associated with obesity in severely obese children. *BMC Med Genet* **11**:. https://doi.org/10.1186/1471-2350-11-58

112. Fang H., Y. Li, S. Du, *et al.* 2010. Variant rs9939609 in the FTO gene is associated with body mass index among Chinese children. *BMC Med Genet* **11**:. https://doi.org/10.1186/1471-2350-11-136

113. Keller K.L., A. Reid, M.C. MacDougall, *et al.* 2010. Sex differences in the effects of inherited bitter thiourea sensitivity on body weight in 4-6-year-old children. *Obesity (Silver Spring)* **18**: 1194–1200. https://doi.org/10.1038/OBY.2009.306

114. Rigoli L., C. Munafò, C. Di Bella, *et al.* 2010. Molecular analysis of the CART gene in overweight and obese Italian children using family-based association methods. *Acta Paediatr* **99**: 722–726. https://doi.org/10.1111/J.1651-2227.2010.01709.X

115. Mong J.L.Y., M.C.Y. Ng, G.S. Guldan, *et al.* 2010. Associations of the growth hormone receptor (GHR) gene polymorphisms with adiposity and IGF-I activity in adolescents. *Clin Endocrinol (Oxf)* **73**: 313–322. https://doi.org/10.1111/J.1365-2265.2010.03786.X

116. Lee H.J., I. kyoung Kim, J.H. Kang, *et al.* 2010. Effects of common FTO gene variants associated with BMI on dietary intake and physical activity in Koreans. *Clin Chim Acta* **411**: 1716–1722. https://doi.org/10.1016/J.CCA.2010.07.010

117. Riestra P., A. Garcia-Anguita, E. Viturro, *et al.* 2010. Influence of the leptin G-2548A polymorphism on leptin levels and anthropometric measurements in healthy Spanish adolescents. *Ann Hum Genet* **74**: 335–339. https://doi.org/10.1111/J.1469-1809.2010.00586.X

118. Pigeyre M., S. Bokor, M. Romon, *et al.* 2010. Influence of maternal educational level on the association between the rs3809508 neuromedin B gene polymorphism and the risk of obesity in the HELENA study. *Int J Obes (Lond)* **34**: 478–486. https://doi.org/10.1038/IJO.2009.260

119. Szopa M., A. Meirhaeghe, J. Luan, *et al.* 2010. No association between polymorphisms in the INSIG1 gene and the risk of type 2 diabetes and related traits. *Am J Clin Nutr* **92**: 252–257. https://doi.org/10.3945/AJCN.2010.29422

120. Den Hoed M., U. Ekelund, S. Brage, *et al.* 2010. Genetic susceptibility to obesity and related traits in childhood and adolescence: influence of loci identified by genome-wide association studies. *Diabetes* **59**: 2980–2988. https://doi.org/10.2337/DB10-0370

121. Bokor S., V. Legry, A. Meirhaeghe, *et al.* 2010. Single-nucleotide polymorphism of CD36 locus and obesity in European adolescents. *Obesity (Silver Spring)* **18**: 1398–1403. https://doi.org/10.1038/OBY.2009.412

122. Wang D., J. Ma, S. Zhang, *et al.* 2010. Association of the MC4R V103I polymorphism with obesity: a Chinese case-control study and meta-analysis in 55,195 individuals. *Obesity (Silver Spring)* **18**: 573–579. https://doi.org/10.1038/OBY.2009.268

123. Vrang N., D. Meyre, P. Froguel, *et al.* 2010. The imprinted gene neuronatin is regulated by metabolic status and associated with obesity. *Obesity (Silver Spring)* **18**: 1289–1296. https://doi.org/10.1038/OBY.2009.361

124. Liem E.T., J.M. Vonk, P.J.J. Sauer, *et al.* 2010. Influence of common variants near INSIG2, in FTO, and near MC4R genes on overweight and the metabolic profile in adolescence: the TRAILS (TRacking Adolescents’ Individual Lives Survey) Study. *Am J Clin Nutr* **91**: 321–328. https://doi.org/10.3945/AJCN.2009.28186

125. Morandi A., C. Maffeis, S. Lobbens, *et al.* 2010. Early detrimental metabolic outcomes of rs17300539-A allele of ADIPOQ gene despite higher adiponectinemia. *Obesity (Silver Spring)* **18**: 1469–1473. https://doi.org/10.1038/OBY.2009.403

126. Kring S.I.I., C. Holst, S. Toubro, *et al.* 2010. Common variants near MC4R in relation to body fat, body fat distribution, metabolic traits and energy expenditure. *Int J Obes (Lond)* **34**: 182–189. https://doi.org/10.1038/IJO.2009.215

127. Moleres A., T. Rendo-Urteaga, C. Azcona, *et al.* 2009. Il6 gene promoter polymorphism (-174G/C) influences the association between fat mass and cardiovascular risk factors. *J Physiol Biochem* **65**: 405–413. https://doi.org/10.1007/BF03185936

128. Hu P., Y.H. Qin, C.X. Jing, *et al.* 2009. Effect of apolipoprotein B polymorphism on body mass index, serum protein and lipid profiles in children of Guangxi, China. *Ann Hum Biol* **36**: 411–420. https://doi.org/10.1080/03014460902882475

129. Pyrzak B., A. Wisniewska, A. Kucharska, *et al.* 2009. No association of LEPR Gln223Arg polymorphism with leptin, obesity or metabolic disturbances in children. *Eur J Med Res* **14 Suppl 4**: 201–204. https://doi.org/10.1186/2047-783X-14-S4-201

130. Johansson L.E., L.M. Johansson, P. Danielsson, *et al.* 2009. Genetic variance in the adiponutrin gene family and childhood obesity. *PLoS One* **4**:. https://doi.org/10.1371/JOURNAL.PONE.0005327

131. Lappalainen S., R. Voutilainen, P. Utriainen, *et al.* 2009. Genetic variation of FTO and TCF7L2 in premature adrenarche. *Metabolism* **58**: 1263–1269. https://doi.org/10.1016/J.METABOL.2009.03.025

132. Popko K., E. Gárska, B. Pyrzak, *et al.* 2009. Influence of proinflammatory cytokine gene polymorphism on childhood obesity. *Eur J Med Res* **14 Suppl 4**: 59–62. https://doi.org/10.1186/2047-783X-14-S4-59

133. Dedoussis G. V., N. Vidra, J. Butler, *et al.* 2009. Peroxisome proliferator-activated receptor-gamma (PPARgamma) Pro12Ala polymorphism and risk for pediatric obesity. *Clin Chem Lab Med* **47**: 1047–1050. https://doi.org/10.1515/CCLM.2009.242

134. Morandi A., L. Pinelli, A. Petrone, *et al.* 2009. The Q121 variant of ENPP1 may protect from childhood overweight/obesity in the Italian population. *Obesity (Silver Spring)* **17**: 202–206. https://doi.org/10.1038/OBY.2008.470

135. Cauchi S., F. Stutzmann, C. Cavalcanti-Proença, *et al.* 2009. Combined effects of MC4R and FTO common genetic variants on obesity in European general populations. *J Mol Med (Berl)* **87**: 537–546. https://doi.org/10.1007/S00109-009-0451-6

136. Vimaleswaran K.S., P.W. Franks, S. Brage, *et al.* 2009. Absence of association between the INSIG2 gene polymorphism (rs7566605) and obesity in the European Youth Heart Study (EYHS). *Obesity (Silver Spring)* **17**: 1453–1457. https://doi.org/10.1038/OBY.2008.650

137. Eisenmann J.C., M.A. Sarzynski, K. Glenn, *et al.* 2009. ACE I/D genotype, adiposity, and blood pressure in children. *Cardiovasc Diabetol* **8**:. https://doi.org/10.1186/1475-2840-8-14

138. Böttcher Y., H. Unbehauen, N. Klöting, *et al.* 2009. Adipose tissue expression and genetic variants of the bone morphogenetic protein receptor 1A gene (BMPR1A) are associated with human obesity. *Diabetes* **58**: 2119–2128. https://doi.org/10.2337/DB08-1458

139. Garcia E.A., B. Heude, C.J. Petry, *et al.* 2008. Ghrelin receptor gene polymorphisms and body size in children and adults. *J Clin Endocrinol Metab* **93**: 4158–4161. https://doi.org/10.1210/JC.2008-0366

140. Jacobsson J.A., P. Danielsson, V. Svensson, *et al.* 2008. Major gender difference in association of FTO gene variant among severely obese children with obesity and obesity related phenotypes. *Biochem Biophys Res Commun* **368**: 476–482. https://doi.org/10.1016/J.BBRC.2008.01.087

141. Grant S.F.A., M. Li, J.P. Bradfield, *et al.* 2008. Association analysis of the FTO gene with obesity in children of Caucasian and African ancestry reveals a common tagging SNP. *PLoS One* **3**:. https://doi.org/10.1371/JOURNAL.PONE.0001746

142. Lagou V., R.A. Scott, Y. Manios, *et al.* 2008. Impact of peroxisome proliferator-activated receptors gamma and delta on adiposity in toddlers and preschoolers in the GENESIS Study. *Obesity (Silver Spring)* **16**: 913–918. https://doi.org/10.1038/OBY.2008.1

143. Wang H.J., H. Zhang, S.W. Zhang, *et al.* 2008. Association of the common genetic variant upstream of INSIG2 gene with obesity related phenotypes in Chinese children and adolescents. *Biomed Environ Sci* **21**: 528–536. https://doi.org/10.1016/S0895-3988(09)60013-1

144. Ochoa M.C., J.L. Santos, C. Azcona, *et al.* 2007. Association between obesity and insulin resistance with UCP2-UCP3 gene variants in Spanish children and adolescents. *Mol Genet Metab* **92**: 351–358. https://doi.org/10.1016/J.YMGME.2007.07.011

145. Körner A., L. Ma, P.W. Franks, *et al.* 2007. Sex-specific effect of the Val1483Ile polymorphism in the fatty acid synthase gene (FAS) on body mass index and lipid profile in Caucasian children. *Int J Obes (Lond)* **31**: 353–358. https://doi.org/10.1038/SJ.IJO.0803428

146. Siddiq A., M. Gueorguiev, C. Samson, *et al.* 2007. Single nucleotide polymorphisms in the neuropeptide Y2 receptor (NPY2R) gene and association with severe obesity in French white subjects. *Diabetologia* **50**: 574–584. https://doi.org/10.1007/S00125-006-0555-2

147. Tobias J.H., C.D. Steer, C. Vilariňo-Güell, *et al.* 2007. Effect of an estrogen receptor-alpha intron 4 polymorphism on fat mass in 11-year-old children. *J Clin Endocrinol Metab* **92**: 2286–2291. https://doi.org/10.1210/JC.2006-2447

148. Körner A., L. Ma, P.W. Franks, *et al.* 2007. Sex-specific effect of the Val1483Ile polymorphism in the fatty acid synthase gene (FAS) on body mass index and lipid profile in Caucasian children. *Int J Obes (Lond)* **31**: 353–358. https://doi.org/10.1038/SJ.IJO.0803428

149. Ghoussaini M., V. Vatin, C. Lecoeur, *et al.* 2007. Genetic study of the melanin-concentrating hormone receptor 2 in childhood and adulthood severe obesity. *J Clin Endocrinol Metab* **92**: 4403–4409. https://doi.org/10.1210/JC.2006-2316

150. Böttcher Y., A. Körner, T. Reinehr, *et al.* 2006. ENPP1 variants and haplotypes predispose to early onset obesity and impaired glucose and insulin metabolism in German obese children. *J Clin Endocrinol Metab* **91**: 4948–4952. https://doi.org/10.1210/JC.2006-0540

151. Herbert A., N.P. Gerry, M.B. McQueen, *et al.* 2006. A common genetic variant is associated with adult and childhood obesity. *Science* **312**: 279–283. https://doi.org/10.1126/SCIENCE.1124779

152. Vogels N., D.L.A. Posthumus, E.C.M. Mariman, *et al.* 2006. Determinants of overweight in a cohort of Dutch children. *Am J Clin Nutr* **84**: 717–724. https://doi.org/10.1093/AJCN/84.4.717

153. Ochoa M.C., M.J. Moreno-Aliaga, M.A. Martínez-González, *et al.* 2006. TV watching modifies obesity risk linked to the 27Glu polymorphism of the ADRB2 gene in girls. *Int J Pediatr Obes* **1**: 83–88. https://doi.org/10.1080/17477160600650386

154. Li S., W. Chen, S.R. Srinivasan, *et al.* 2006. Influence of lipoprotein lipase gene Ser447Stop and beta1-adrenergic receptor gene Arg389Gly polymorphisms and their interaction on obesity from childhood to adulthood: the Bogalusa Heart Study. *Int J Obes (Lond)* **30**: 1183–1188. https://doi.org/10.1038/SJ.IJO.0803281

155. Marti A., M.C. Ochoa, A. Sánchez-Villegas, *et al.* 2006. Meta-analysis on the effect of the N363S polymorphism of the glucocorticoid receptor gene (GRL) on human obesity. *BMC Med Genet* **7**:. https://doi.org/10.1186/1471-2350-7-50
