# Supplementary material for: Genetics of Common Obesity in Children and Adolescents
Source: Ann N Y Acad Sci. 2025 Oct 13;1553(1):34–49. doi: 10.1111/nyas.70070 (PMC12645270; doi:10.1111/nyas.70070)
Supplement: Supplementary file 2 — Supplementary Material: Appendix 2 [file NYAS-1553-34-s002.docx]

Appendix 2. Quality assessment of GWAs and meta-analyses on childhood obesity and related phenotypes according to STREGA

| **Study** | **Quality** |
| --- | --- |
| Chatterjee S et al., 2021 | High |
| Vogelezang S et al., 2020 | Medium |
| Costa-Urrutia et al., 2019 | Medium |
| Zhao et al., 2019 | Low |
| Bradfield et al., 2019 | High |
| Felix et al., 2016 | Low |
| Hwang et al., | Medium |
| Warrington et al., 2015 | High |
| Graff et al., 2013 | High |
| Wang et al., 2012 | Medium |
| Bradfield et al., 2012 | Low |
| Comuzzie AG et al., 2012 | Medium |
| Zhao J et al., 2011 | Medium |
| Zhao et al., 2009 | Medium |
| Frayling TM et al., 2007 | Medium |
| Dina C et al., 2007 | Low |
